# Quantification of HER2 and estrogen receptor heterogeneity in breast cancer by single-molecule RNA fluorescence in situ hybridization

**DOI:** 10.18632/oncotarget.15727

**Published:** 2017-02-25

**Authors:** Laura Annaratone, Michele Simonetti, Erik Wernersson, Caterina Marchiò, Silvano Garnerone, Maria Stella Scalzo, Magda Bienko, Roberto Chiarle, Anna Sapino, Nicola Crosetto

**Affiliations:** ^1^ Department of Medical Sciences, University of Turin, Turin, Italy; ^2^ Department of Medical Biochemistry and Biophysics, Science for Life Laboratory, Division of Translational Medicine and Chemical Biology, Karolinska Institutet, Stockholm, Sweden; ^3^ Department of Laboratory Medicine, Pathology Unit, Azienda Ospedaliera Città della Salute e della Scienza di Torino, Turin, Italy; ^4^ Department of Pathology, Boston Children’s Hospital, Harvard Medical School, Boston, Massachusetts, USA; ^5^ Department of Molecular Biotechnology and Health Sciences, University of Turin, Turin, Italy; ^6^ Candiolo Cancer Institute – FPO, IRCCS, Candiolo, Italy

**Keywords:** single-molecule RNA FISH (smFISH), breast cancer, intra-tumor heterogeneity, epidermal growth factor receptor 2 (HER2), estrogen receptor 1 (ER), Pathology Section

## Abstract

Intra-tumor heterogeneity is a pervasive property of human cancers that poses a major clinical challenge. Here, we describe the characterization, at the transcriptional level, of the intra-tumor topography of two prominent breast cancer biomarkers and drug targets, epidermal growth factor receptor 2 (HER2) and estrogen receptor 1 (ER) in 49 archival breast cancer samples. We developed a protocol for single-molecule RNA FISH in formalin-fixed, paraffin-embedded tissue sections (FFPE-smFISH), which enabled us to simultaneously detect and perform absolute quantification of HER2 and ER mature transcripts in single cells and multiple tumor regions. We benchmarked our method with standard diagnostic techniques, demonstrating that FFPE-smFISH is able to correctly classify breast cancers into well-established molecular subgroups. By counting transcripts in thousands of single cells, we identified different expression modes and levels of inter-cellular variability. In samples expressing both HER2 and ER, many cells co-expressed both genes, although expression levels were typically uncorrelated. Finally, we applied diversity metrics from the field of ecology to assess the intra-tumor topography of HER2 and ER gene expression, revealing that the spatial distribution of these key biomarkers can vary substantially even among breast cancers of the same subtype. Our results demonstrate that FFPE-smFISH is a reliable diagnostic assay and a powerful method for quantification of intra-tumor transcriptional heterogeneity of selected biomarkers in clinical samples.

## INTRODUCTION

Intra-tumor heterogeneity (ITH) is a hallmark of human cancers that manifests itself at the genetic, epigenetic, and phenotypic level [1]. From the clinical standpoint, ITH of actionable mutations and more generally of any type of molecular biomarker is a major challenge, as the fraction of tumor cells expressing the mutation or biomarker of interest and possibly their geographical distribution within the tumor will influence therapeutic response. Moreover, the spatial organization of different subclones - and not only their number - is likely to affect clinical outcome [2].

In the past few years, multi-region sequencing [3] and single-cell sequencing [4] technologies have revealed that ITH is prevalent in many cancer types. An important limitation of these approaches, however, is their intrinsic inability to provide information about the spatial arrangement of different subclones and the topography of cells expressing selected biomarkers. In contrast, in spite of the typically much lower throughput compared to sequencing technologies, *in situ* methods such as fluorescence *in situ* hybridization (FISH) can provide more robust quantitative information not only on the abundance, but also on the location of selected DNA and RNA targets inside their cellular and tissue context. For example, combined DNA FISH and immunostaining were used to study the spatial organization of different subclones in breast cancer samples obtained before and after neoadjuvant chemotherapy [5], and to compare genetic and phenotypic ITH in breast cancer metastases and matched primary lesions [6]. More recently, a novel *in situ* method named STAR-FISH was successfully applied, together with DNA FISH, to probe the temporal and spatial heterogeneity of PIK3CA mutations and HER2 amplification in HER2-positive breast cancers treated with neoadjuvant therapy [7]. On the RNA side, single-molecule RNA FISH (smFISH) [8] and RNAscope [9] have emerged as powerful methods enabling visualization, precise localization, and enumeration of individual RNA molecules within fixed cells and tissues (for a detailed review of these two methods, see for example ref. [10]). Padlock probes and rolling circle amplification have also been used to visualize RNA molecules in fixed cells and tissues [11]. Additionally, rolling circle amplification was recently applied to quantify spatial ITH of clinically relevant KRAS, EGFR and TP53 mutations in lung cancer [12]. Recently, we applied smFISH to quantify the intra-tumor transcriptional heterogeneity of the oncogenic fusion gene, BCR-ABL, in chronic myeloid leukemia [13], and to study the cell-to-cell variability of immunoglobulin gene expression in follicular lymphoma cell lines [14]. However, application of smFISH to clinical tissue samples, including formalin-fixed, paraffin-embedded (FFPE) specimens, has been so far very limited. Moreover, a direct and rigorous comparison of smFISH with routine diagnostic methods has not been done yet. Here, we developed and validated a robust protocol for smFISH in FFPE breast cancer tissue sections (FFPE-smFISH), and applied it to quantify transcriptional ITH and to perform spatial analysis, at single-cell level, of the two most clinically relevant breast cancer biomarkers: epidermal growth factor receptor 2 (HER2) and estrogen receptor 1 (ER).

## RESULTS

### Development of a HER2 and ER FFPE-smFISH assay for nascent and mature transcripts

**Figure 1:**
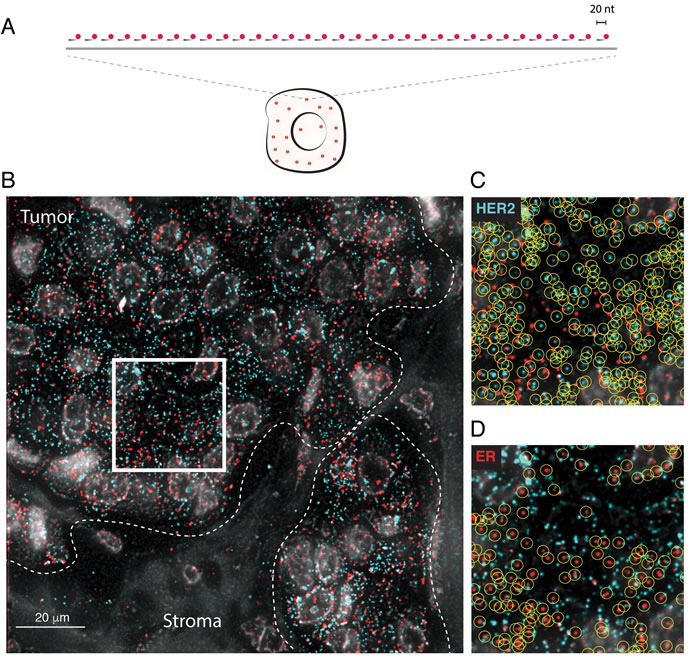
FFPE-smFISH **A**. Scheme of smFISH. A pool of usually 30-50 oligos, each of 20 nucleotides (nt) length and labeled with a single fluorophore (red), is hybridized *in situ* to a complementary target RNA (gray line). As a rule of thumb, a transcript that can be targeted with at least 20 oligos can be detected by smFISH. **B**. Example of HER2 (cyan) and ER (red) mRNA detection in a FFPE breast cancer tissue section. Each spot corresponds to a diffraction-limited fluorescent signal that is generated upon binding of the fluorescently labeled oligos forming the smFISH probe to their target. Gray, DAPI-stained nuclei. The white dashed lines mark the boundary between tumor and stroma. Tumor regions are typically characterized by a high density of large nuclei with peripheral chromatin, whereas the nuclear density is much lower in stromal areas. (C-D) Magnification of the region encircled by the white square in **B**. **C**. HER2 transcripts (cyan dots). **D**. ER transcripts (red dots). The yellow circles mark individual mRNA molecules automatically identified by our custom software.

In smFISH, individual transcripts are targeted by probes consisting of pools of 20 nucleotides (nt)-long complementary oligonucleotides, each conjugated to a single fluorophore, and are detected under a wide-field epifluorescence microscope as bright diffraction-limited spots that can be precisely enumerated and localized inside single cells (Figure 1A). In order to design smFISH probes targeting all the main isoforms of human HER2 and ER genes, we took advantage of our recently developed probe database that covers all the protein-coding transcripts annotated in Ensembl (see www.fusefish.eu [13]). We managed to cover each transcript with at least 48 oligos, which is sufficient to yield a robust smFISH signal, as previously demonstrated [8]. A full list of oligo sequences is available in Supplementary Table 1. Initially, we only applied standard deparaffinization of the FFPE tissue sections before performing smFISH. However, high background autofluorescence prevented robust smFISH signal detection (data not shown). After several trials, we identified an optimal pre-hybridization sample processing procedure that includes a post-fixation step in methanol-acetic acid, and two RNA retrieval steps to partially reverse cross-links and ensure target accessibility. In addition, we included an autofluorescence-quenching step in sodium borohydride, which was previously applied to detect nascent mRNAs in prostate cancer FFPE biopsies [15]. A step-by-step FFPE-smFISH protocol is provided in Supplementary Information.

We applied our optimized FFPE-smFISH protocol to 49 archival specimens of invasive breast cancer in which the HER2 and ER status had been thoroughly assessed by standard methods. Patients were all females, mostly post-menopausal (95.9% > 50 year-old) and treated with conservative surgery (59.2%, Table 1). Tumors were predominantly invasive ductal carcinomas (79.6%) of high histological grade (47% grade 3) and ER-positive (75.5%). 44.9% of cases were HER2-positive based on immunohistochemistry (IHC) and DNA FISH (Table 2).

**Table 1:** Patient characteristics

Age at diagnosis (Years)	Number of cases (%)
<40	0 (0%)
40-50	2 (4.1%)
51-70	30 (61.2%)
>70	17 (34.7%)
**Sex**	
M	0 (0%)
F	49 (100%)
**Surgery type**	
Mastectomy	20 (40.8%)
Conservative	29 (59.2%)

**Table 2:** Histological and molecular characteristics of tumors analyzed

Histologic type		Number of cases (%)
IDC		39 (79.6%)
ILC		7 (14.3%)
Others		3 (6.1%)
**Histologic grade**		
1		11 (22.4%)
2		14 (28.6%)
3		23 (47%)
Undetermined		1 (2%)
**ER status**		
0		12 (24.5%)
>1%		37 (75.5%)
**HER2 status**		
**IHC score**	**DNA FISH**	
0	Not Amp	10 (20.4%)
	Amp	0 (0%)
1+	Not Amp	7 (14.3%)
	Amp	0 (0%)
2+	Not Amp	10 (20.4%)
	Amp	7 (14.3%)
3+	Not Amp	0 (0%)
	Amp	15 (30.6%)
**Ki67 score**		
> 14%		35 (71.4%)
< 14%		14 (28.6%)
**Molecular Subtype**		
LumA		10 (20.4%)
LumB/HER2-neg		12 (24.5%)
LumB/HER2-pos		15 (30.6%)
Triple-neg		5 (10.2%)
HER2		7 (14.3%)

IDC, invasive ductal carcinoma. ILC, invasive lobular carcinoma. ER, estrogen receptor. IHC, immunohistochemistry. FISH, fluorescence in situ hybridization.

In each sample, we simultaneously imaged HER2 and ER mature and nascent transcripts at high magnification (100X objective) in at least 32 regions (range: 32-79) inside macroscopic tumor areas that a pathologist had previously marked in an adjacent hematoxylin-eosin (H&E) stained section (Figure 1B and Supplementary Table 2). Individual transcripts appeared as bright diffraction-limited spots that could be identified and counted in an automated manner (Figure 1C and 1D, and Materials and Methods). Importantly, HER2 and ER transcripts were localized within regions containing many nuclei displaying morphologic features of cancer cells (as confirmed by a pathologist), while they were largely absent in stromal regions, as exemplified in Figure 1B, and as it can be appreciated by visualizing all the images in the freely accessible supporting website, http://tumorheterogeneity.eu/.

To further confirm that HER2 and ER smFISH signals overlap with tumor regions, we applied to selected cases a new pipeline, which we recently developed in our lab (unpublished data) that enables registration of smFISH images acquired at high magnification (100X) onto a large-field (up to 2 × 2 cm) scan of the same tissue section stained with H&E after smFISH, and imaged at low magnification (10X) (http://tumorheterogeneity.eu/ and Materials and Methods). We managed to detect HER2 and ER transcripts in FFPE blocks as old as 3 years, and in FFPE tissue sections mounted on microscope slides and stored at room temperature up to 4 years, suggesting that FFPE-smFISH might be a robust method for biomarker analysis even in old archival samples.

### FFPE-smFISH scoring

We sought to develop a fast and robust scoring approach to quantify HER2 and ER smFISH signals, which would be easy to apply in the diagnostic setting. For this purpose, we segmented each 1,024 × 1,024 px image (1 px = 125 nm) with a regular grid of squared pseudo-cells, and defined as smFISH score the mean density of each transcript inside all the pseudo-cells of a given case. To choose the size of the pseudo-cells, we first segmented individual cells by using the boundary of nuclei stained with 4′,6-diamidino-2-phenylindole (DAPI) as a reference. In order to account for transcripts localized in the cytoplasm, we uniformly dilated the margins of the segmented nuclei by a constant length (Materials and Methods).

We first tested the reproducibility of the single-cell segmentation approach by segmenting DAPI-stained nuclei in three replica samples of three different cell lines expressing variable levels of HER2 (Supplementary Figure 1A). We then manually segmented 38,191 cells (1,060 ± 699 cells per case, mean ± s.d.) in 36 out of the 49 cases. On average, we analyzed 57 images per case (57 ± 11, mean ± s.d.) and segmented 19 cells per image (19 ± 10, mean ± s.d., Supplementary Table 2). Next, we computed the area of the segmented cells at different nucleus dilation lengths, and selected different pseudo-cell sizes so that the area of the pseudo-cells overlaps with the interquartile range of the area of the segmented cells at different dilation lengths (Supplementary Figure 1B). We calculated the Spearman correlation between differently sized pseudo-cells and nucleus dilation lengths using four different thresholds of the number of smFISH dots per pseudo- or segmented cell (≥ 0, 1, 2, 3). Both in the case of HER2 and ER, we observed a very strong correlation (Spearman ρ > 0.9 for HER2 and ρ > 0.85 for ER, *P* < 0.001) between the smFISH scores calculated based on pseudo- *vs*. segmented cells (Supplementary Figure 1C and 1D). In addition, we developed a segmentation algorithm that uses the spatial distribution of DAPI intensity to find cell-containing regions inside each image (Supplementary Figure 1E and Materials and Methods). As for segmented cells, we found a very strong correlation (Spearman ρ > 0.95 for HER2 and ρ > 0.84 for ER, *P* < 0.001) between the mRNA density per pseudo-cell and the density inside the regions identified by our algorithm (Supplementary Figure 1F and 1G). Altogether, these results demonstrate that the pseudo-cell segmentation approach is a robust method to score smFISH signals that, while retaining information about inter-regional differences, is much faster than single-cell segmentation and is therefore ideal in the diagnostic setting.

### Assay validation and reproducibility

**Figure 2:**
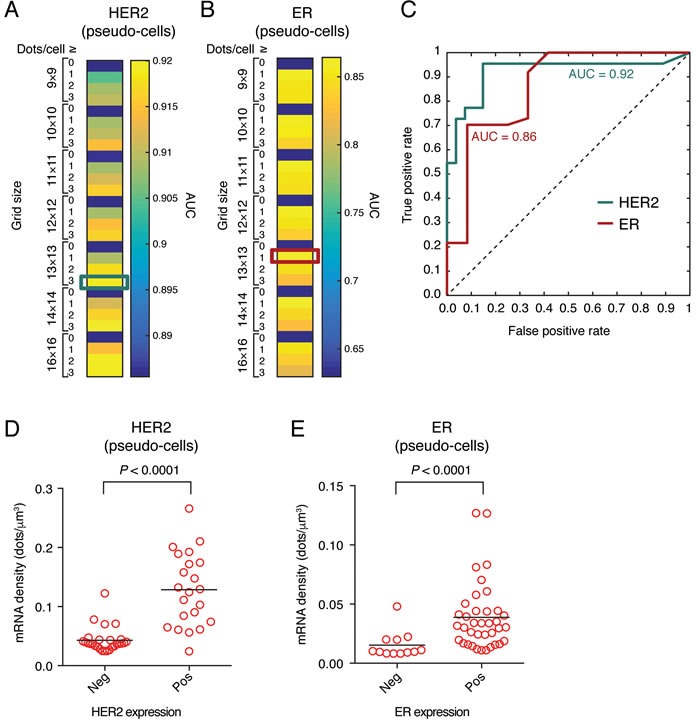
Diagnostic performance of HER2 and ER FFPE-smFISH **A**. Heatmap showing different values of the HER2 area under the curve (AUC) obtained for different pseudo-cell sizes and thresholds of the number of mRNA dots per cell based on the ROC curve method (see Materials and Methods). Cyan box, pseudo-cell and threshold combination yielding the HER2 ROC curve shown in **C**., and used in all subsequent pseudo-cell analyses. **B**. Same as in **A**., but for ER. Red box, pseudo-cell and threshold combination yielding the ER ROC curve shown in **C**., and used in all subsequent pseudo-cell analyses. **C**. Diagnostic performance of HER2 and ER FFPE-smFISH obtained with the pseudo-cell size and threshold combinations indicated by the cyan and red boxes in **A**. and **B**., respectively. **D**. Mean HER2 transcript density in tumors categorized as HER2-positive or negative based on IHC and DNA FISH. **E**. Mean ER transcript density in tumors categorized as ER-positive or negative based on IHC. Each dot in **D**. and **E**. corresponds to the mean pseudo-cell transcript density in one of the 49 analyzed cases. Horizontal black bars represent the mean of all cases in the corresponding group. *P* values were obtained with the Mann-Whitney test (two-tailed).

Next, we aimed to systematically validate our method and assess its reproducibility. First, we determined which pseudo-cell size and threshold combination gives the best diagnostic performance, by applying the Receiver Operating Characteristic (ROC) method [16] to compare the pseudo-cell based scores with the IHC and DNA FISH scores determined for the same samples according to the ASCO/CAP guidelines [17] (Materials and Methods). For HER2, the highest diagnostic performance values (AUC > 0.90) were obtained with a threshold of 2-3 dots per pseudo-cell (Figure 2A). In the case of ER, the diagnostic performance was slightly lower (this could be explained by the low ER positivity threshold used to score IHC results, see Materials and Methods), with the highest AUC values ( > 0.85) obtained with a threshold of 1 dot per pseudo-cell (Figure 2B). A grid of 13 × 13 pseudo-cells and a threshold of 3 dots per pseudo-cell for HER2 and 1 dot per pseudo-cell for ER maximized the following three parameters: 1) area of pseudo- *vs*. segmented cells; 2) Spearman correlation coefficient of the mean transcript density in pseudo- *vs*. segmented cells; and 3) diagnostic performance assessed by the ROC method (Figure 2C). Hence, we used these settings in all subsequent analyses based on pseudo-cells.

According to current guidelines, patients with IHC 3+ score and patients with IHC 2+ score and HER2 amplification confirmed by DNA FISH are considered HER2-positive and thus eligible to anti-HER2 therapy [17]. We observed that the amount of HER2 smFISH signals increased with the IHC score, with the highest expression observed in 3+ samples (Supplementary Figure 2A). Accordingly, the mean HER2 transcript density per pseudo-cell determined by FFPE-smFISH (HER2 FFPE-smFISH score) was significantly higher in positive samples (mean = 0.13 *vs*. 0.04 dots/μm^3^, Mann-Whitney test, *P* < 0.001, Figure 2D). Of note, one case (case 21) in the positive group had a very low smFISH score (0.024 dots/μm^3^) confirmed by the three replicate experiments, while ER was expressed in the same case, ruling our RNA degradation. Accordingly, repetition of HER2 IHC in the same sample and re-evaluation of the data by a pathologist revealed a heterogeneous IHC 2+ score with borderline DNA FISH positivity, placing this case rather in the negative group (Supplementary Table 3). We also observed that the mean HER2 score was significantly higher in the IHC 3+ group as compared to the IHC 2+ amplified group (mean HER2 score = 0.15 *vs*. 0.07 dots/μm^3^, Mann-Whitney test, *P* = 0.0021, Supplementary Figure 2B). Although patients in these two tumor groups are currently treated with the same anti-HER2 therapy regimens, it is possible that tumors with higher HER2 mRNA levels as detected by smFISH might respond better to HER2-targeted therapy. In line with this, a significant association between the level of HER2 amplification detected by DNA FISH and overall survival was recently described [18], suggesting that scoring HER2 on a continuous scale might be better than categorical scoring. For 6 selected cases for which additional sections were available, an independent experimenter, in a different laboratory and using a different microscope setup (Materials and Methods) successfully replicated the inter-sample differences observed in our initial screening, demonstrating the reproducibility of our assay (Supplementary Figure 2C). Furthermore, we compared smFISH with an independent RNA-based method - reverse transcription quantitative PCR (RT-PCR) - obtaining a good correlation between the two measurements (Spearman ρ = 0.75, *P* = 0.06, Supplementary Figure 2D and Materials and Methods). Notably, comparison of the clinical samples with the SKBR3 breast cancer cell line, both by smFISH and RT-PCR, demonstrated that, while smFISH has a relatively broad dynamic range and is capable of detecting high mRNA levels, the expression of HER2 in breast cancer cell lines might not reflect the actual transcript abundance in clinical samples, similarly to what we previously observed for the oncogenic fusion transcript, BCR-ABL1, in chronic myeloid leukemia clinical samples and cell lines [13].

In analogy to HER2, the mean ER transcript density per pseudo-cell determined by FFPE-smFISH (ER FFPE-smFISH score) was on average significantly higher in tumors scored positive by IHC (mean ER score = 0.04 *vs*. 0.02 dots/μm^3^, Mann-Whitney test, *P* < 0.001, Figure 2E). However, many tumors expressed mRNA levels comparable to the negative group (Figure 2E), which might at least in part depend on the criteria used for scoring samples as positive and/or on the antibody used for IHC (Materials and Methods). Another possibility is that some of the cases scored positive by IHC express shorter ER isoforms [19, 20] so that a smaller number of smFISH oligos can actually hybridize to the target compared to the full-length ER mRNA, thus resulting in a weaker signal.

Next, we examined if the HER2 and ER FFPE-smFISH scores correlate with the molecular subtype, which was surrogated using the results of IHC markers as described in Materials and Methods. In the case of HER2, Luminal A, Luminal B/HER2-negative, and triple-negative subtypes had low expression levels (mean HER2 score = 0.047, 0.044, and 0.031 dots/μm^3^, respectively), whereas the Luminal B/HER2-positive and HER2 groups had higher scores. The HER2-positive subtype expressed significantly higher levels in comparison to the Luminal B/HER2-positive subtype (mean HER2 score = 0.17 *vs*. 0.11 dots/μm^3^, Mann-Whitney test, *P* = 0.05, Supplementary Figure 3A). In the case of ER, as expected HER2-positive and triple-negative subtypes had the lowest ER scores (mean ER score = 0.02 and 0.01 dots/μm^3^, respectively), whereas Luminal A and Luminal B/HER2-positive samples had the highest scores (mean ER score = 0.042 and 0.044 dots/μm^3^, respectively). Two tumors in the latter group had an ER score three times higher than the mean score of all the cases in the same group (0.12 *vs*. 0.04 dots/μm^3^) although the IHC scores were comparable (Supplementary Figure 3B and Supplementary Table 3).

To further validate our approach, we compared HER2 FFPE-smFISH with three other methods that can assess the HER2 status in clinical breast cancer samples: DNA FISH, Multiplex Ligation-dependent Probe Amplification (MLPA) [21] and Proximity Ligation Assay (PLA) [22]. In line with our previous observations [23], we found a positive correlation between the HER2 DNA copy number assessed by either DNA FISH (Spearman ρ = 0.79, *P* < 0.0001, Supplementary Figure 3C) or MLPA (Spearman ρ = 0.81, *P* < 0.0001, Supplementary Figure 3D) and HER2 mRNA levels. A weaker, but statistically significant correlation was also observed between RNA levels and protein levels determined by PLA (Spearman ρ = 0.63, *P* < 0.0001, Supplementary Figure 3E). Overall, these results demonstrate that pseudo-cell transcript densities measured by FFPE-smFISH are a robust metric of HER2 and ER abundance that may be implemented in routine clinical breast cancer diagnostics.

### Single-cell analysis of HER2 and ER expression

We then asked whether FFPE-smFISH is a valid method to assess the intra-tumor transcriptional heterogeneity of HER2 and ER. For this purpose, we first analyzed in detail the data obtained from the single-cell segmentation approach described above (Supplementary Figure 1B). The distribution of pairwise distances between all segmented cells in each image was similar in all the segmented cases (coefficient of variation of the means = 4%), indicating that the manual segmentation was performed homogeneously (Supplementary Figure 4A). We observed that in triple-negative, HER2-positive, and Luminal B/HER2-positive tumors the nuclei were on average slightly, but significantly larger in comparison to Luminal A and Luminal B/HER2-negative samples (75.47 *vs*. 63.84 μm^2^, Mann-Whitney test, *P* = 0.019, Supplementary Figure 4B), possibly reflecting the difference in biological and clinical aggressiveness between these subtypes. Next, we again applied ROC analysis in order to choose the nucleus expansion margin and threshold of dots per cell to apply in subsequent analyses. As shown in Supplementary Figure 4C-E, an expansion margin of 20 px (2.5 μm) and a threshold of 0 dots per cell in the case of HER2 and 1 dot per cell in the case of ER, gave the highest diagnostic performance. Thus, we used these settings in all subsequent single-cell analyses.

**Figure 3:**
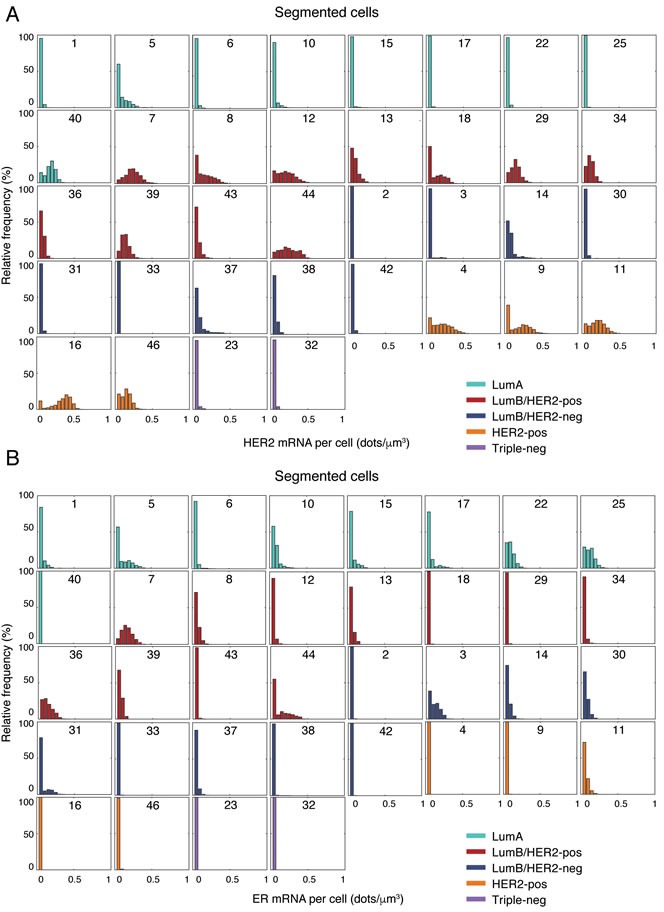

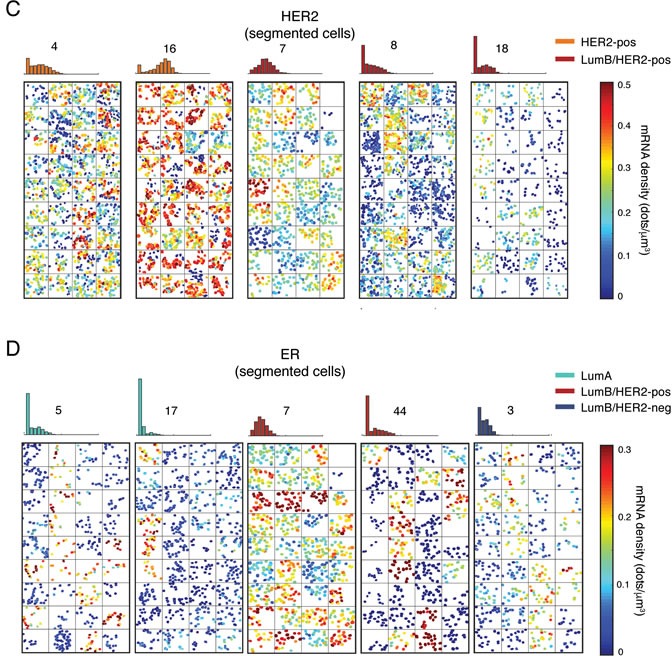
HER2 and ER gene expression in single cells **A**. Distribution of the HER2 mRNA density per cell in 36 segmented cases grouped by molecular subtype. The number in each box indicates the case ID (see Supplementary Table 3). **B**. Same as in **A**., but for ER. **C**. Representative spatial HER2 mRNA patterns in selected cases belonging to the HER2-positive or Luminal B/HER2-positive molecular subtype. For each case, we randomly selected 36 images and plotted all the cells segmented in each image (nucleus expansion margin = 20 px; threshold = 0 dots/cell) as polygons filled with a different color depending on the mRNA density in the cell. Finally, we arranged the plots in a 9 × 4 matrix plot, as shown here. Therefore, adjacent plots in the matrix must not be interpreted as coming from physically adjacent regions in the tumor. The histogram on top of each matrix shows the distribution of mRNA density among all the cells in the case, independently of their location (same as in **A**. and **B**.). The number above each histogram represents the case ID (see Supplementary Table 3). The color bar on the right shows the colors encoding for the mRNA density in each cell. **D**. Same as in **C**., but for ER. Similar maps for all the 36 segmented cases can be browsed on the supporting website, http://tumorheterogeneity.eu/.

Next, we plotted the single-cell distribution of HER2 and ER expression in all the segmented tumors grouped according to their molecular subtype (Figure 3A-3B). As expected, HER2 was very lowly expressed in triple-negative, as well as in most Luminal B/HER2-negative tumors (HER2 score ≤ 0.05 dots/μm^3^). However, two cases in the latter group (case 14 and 37) featured a small sub-population of highly expressing cells (~5% of cells with HER2 score ≥ 0.3 dots/μm^3^) coexisting with a much larger population of lowly expressing cells ( > 50% of cells with HER2 score ≤ 0.05 dots/μm^3^) despite the fact that these tumors were diagnosed as HER2-negative by IHC. Indeed, re-evaluation of the IHC data by a pathologist identified two different populations of tumor cells (30% of cells with HER2 score 2+ and 70% with score 1+ in case 14, see Supplementary Table 3). Among Luminal A tumors, case 5 had a small fraction of high-expressing cells (~5% of cells with HER2 score ≥ 0.3 dots/μm^3^), while the majority of cells in case 40 expressed HER2 at levels similar to the HER2-positive cases (~50% of cells with HER2 score ≥ 0.2 dots/μm^3^). In some cases (case 8, 9, and 18) we observed a clear transcriptionally ‘off’ population of cells ( > 30% of cells with HER2 score ≤ 0.05 dots/μm^3^) together with an ‘on’ population of cells expressing higher levels of HER2 mRNA (HER2 score ≥ 0.2 dots/μm^3^). In the case of ER, ER mRNA was, as expected, almost undetectable in the triple-negative and HER2-positive tumors analyzed (ER score ≤ 0.05 dots/μm^3^). However, single-cell expression levels were also markedly low in one Luminal A (case 40, 100% of cells with ER score ≤ 0.05 dots/μm^3^), as well as in several Luminal B tumors (case 2, 18, 29, 33, 38, 42, and 43, > 95% of cells with ER score ≤ 0.05 dots/μm^3^). In some cases, cells expressing low ER levels coexisted with cells expressing moderate-to-high levels (ER score ≥ 0.2 dots/μm^3^), while some tumors (case 5, 31, and 44) showed one transcriptionally ‘off’ population of cells ( > 50% of cells with ER score ≤ 0.05 dots/μm^3^) together with an ‘on’ population of cells expressing higher levels of ER mRNA (ER score ≥ 0.1 dots/μm^3^), which was also detected at the protein level by careful re-evaluation of the IHC images by a pathologist (Supplementary Table 3).

To further investigate our sample cohort at the single-cell level, we used heatmap plots to visualize the spatial distribution of the density of HER2 and ER transcripts in the segmented cells inside each field of view (Figure 3C and 3D and http://tumorheterogeneity.eu/). To quantify the inter-regional variability of the spatial distribution of transcript densities (i.e., the difference between all the fields of view in a tumor), we computed the mean transcript density per cell per field of view in HER2-positive (for HER2) and ER-positive (for ER) tumors. On average, the inter-regional variability was higher in the case of ER than HER2 (mean coefficient of variation = 0.84 *vs*. 0.44, Mann-Whitney test, *P* < 0.0001, Supplementary Figure 5A). Interestingly, even in the same molecular subtype, we observed different spatial patterns, with some cases displaying homogeneously high expression levels in all the fields of view (e.g., HER2 in case 16), other cases with an admixture of lowly and highly expressing cells in all the images (e.g., HER2 in case 4), and other cases with local homogeneity (either low or high expression) and global heterogeneity (‘cold’ and ‘hot’ fields, see for example HER2 and ER in case 7). Notably, in some bimodal cases the ‘on’ and ‘off’ populations were spatially segregated (e.g., HER2 in case 8 and ER in case 44), whereas in others there was more promiscuity between ‘on’ and ‘off’ cells within the same field of view (e.g., HER2 in case 18, and ER in case 5 and 17) (Figure 3C and 3D). Importantly, careful re-assessment of all the cases displaying bimodality by a pathologist confirmed that these transcriptionally silent cells are indeed localized inside tumor regions and have a nuclear morphology compatible with tumor cells (see corresponding images in the supporting website, http://tumorheterogeneity.eu/), thus excluding that the ‘off’ populations observed come from stroma. We also wondered whether in tumors with broad or bimodal HER2 or ER expression we would detect a morphological difference between cells expressing low versus high transcript levels, following the recent observation that tumors carrying a high mutational burden and high levels of copy number alterations have on average larger nuclei [24]. In the case of HER2, we found that highly expressing cells had slightly, but statistically significantly larger nuclei (68.31 *vs*. 65 μm^2^, Mann-Whitney test, *P* < 0.0001, Supplementary Figure 5B), whereas we observed the opposite for ER (62.60 *vs*. 65.17 μm^2^, Mann-Whitney test, *P* = 0.0007, Supplementary Figure 5C). Altogether, these results indicate that, although the average expression level of HER2 and ER may be similar among breast cancers of the same molecular subtype, the spatial distribution and expression level of single tumor cells may substantially vary, which in turn might influence responsiveness to therapy.

### Correlation between HER2 and ER transcript levels in single cells

**Figure 4:**
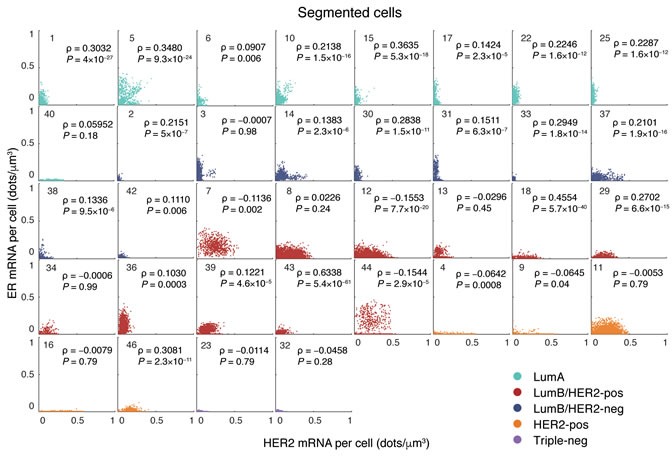
Single-cell correlation between HER2 and ER mRNA density in all the 36 segmented tumors grouped by molecular subtype Each dot in the scatter plots represents a single cell. ρ, Spearman correlation. *P* values were obtained with the Student's t test (two-tailed).

Clinical and laboratory evidence indicate that the cross-talk between HER2 and ER signaling pathways has a critical role in mediating the response to endocrine therapy [25, 26]. Since smFISH allows simultaneous visualization of two transcripts in the same cell, we checked whether a co-dependency between HER2 and ER mRNA levels could be detected and serve as a proxy of the crosstalk between the two pathways. While in the majority of cases we found no significant correlation between the HER2 and ER expression levels in single cells (Spearman ρ < 0.1, *P* > 0.05), in some cases there was a weak, but statistically significant positive correlation (0.2 < ρ < 0.4, *P* < 0.05, Figure 4). Interestingly, in the two Luminal A cases that were found to express relatively high levels of HER2 mRNA (case 5 and 10, see Figure 3A), there was a small, but clearly visible subpopulation of cells with positively correlated levels of HER2 and ER mRNA (Spearman ρ = 0.35, *P* = 10^−24^ for in case 5, and ρ = 0.21, *P* = 10^−16^ in case 10, Figure 4). These results demonstrate the ability of FFPE-smFISH to pinpoint differences in the population structure of a tumor that might have important biological and clinical consequences, but that would go undetected with standard diagnostic techniques.

### Intra-tumor topography of HER2 and ER expression

**Figure 5:**
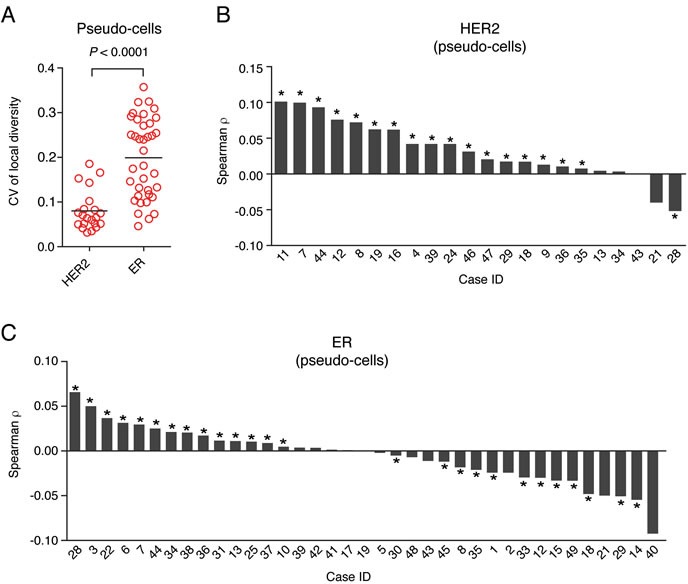
Spatial heterogeneity of HER2 and ER gene expression **A**. Coefficient of variation (CV) of HER2 and ER local diversity scores calculated for each field of view in HER2-positive and ER-positive cases, respectively (in the HER2 group case 21 was excluded due to the low number of tumor regions that were imaged). Each dot represents the CV of the vector of local diversity index values obtained for all the fields of view of each case. Horizontal black bars represent the mean of all cases in the corresponding group. The *P* value was obtained with the Mann-Whitney test (two-tailed). **B**. Waterfall plot of the Spearman correlation between the difference in HER2 mRNA density and the Euclidean distance separating two pseudo-cells in the same field of view in HER2-positive cases. **D**. Same as in **C**., but for ER. The asterisks indicate statistically significant correlations (*P* < 0.05, Student's t test, two-tailed).

We then sought to characterize the spatial heterogeneity of HER2 and ER in a more systematic way. For this purpose, we adapted several diversity metrics that were previously applied to characterize genetic ITH in breast cancer [5, 6]. First, we used the Shannon entropy - a metric originally developed in information theory and used, for example, to characterize the diversity of species in an ecosystem - to compare how the HER2 and ER expression levels vary among all the pseudo-cells in the same field of view (local diversity index) or in the whole tumor (global diversity index) (Materials and Methods). The local diversity index indicates how many different expression levels are detected inside each imaged tumor region, whereas the global entropy reflects how many different expression levels are overall present in all the tumor regions analyzed. For both HER2 and ER, the mean local and global diversity indexes were positively correlated, but the local values were typically higher (Supplementary Figure 6A-D). In the case of ER, the global diversity was significantly lower than the mean local diversity and more heterogeneous among different cases (mean = 0.69 *vs*. 0.57, Mann-Whitney test, *P* = 0.0009, Supplementary Figure 6D). On average, the mean local diversity was higher in the case of HER2 than for ER (mean = 0.85 *vs*. 0.69, Supplementary Figure 6C and 6D), but interestingly the ER local diversity index was significantly more variable from region to region of the same tumor (mean coefficient of variation = 0.2 for ER *vs*. 0.08 for HER2, Mann-Whitney test, *P* < 0.0001, Figure 5A), possibly reflecting differences in the mutational or dispersal rates of different sub-clones expressing various levels of HER2 and ER. Molecular subtype HER2-positive tumors had on average a significantly higher local diversity compared to Luminal B/HER2-positive tumors (mean = 0.87 *vs*. 0.84, Mann-Whitney test, *P* = 0.045, Supplementary Figure 6E), whereas there was no significant difference in the ER local diversity among different luminal subtypes (Supplementary Figure 6F).

We then analyzed the difference in mRNA levels between adjacent pseudo-cells (Materials and Methods). Both for HER2 and ER, the mean mRNA density per pseudo-cell and the difference in transcript counts between adjacent pseudo-cells were strongly positively correlated (Spearman ρ = 0.86 for HER2 and ρ = 0.95 for ER, *P* < 0.0001, Supplementary Figure 7A-B). In both cases, we also found a weaker, but statistically significant positive correlation between the mean difference in transcript levels among adjacent pseudo-cells and the average local diversity index (Spearman ρ = 0.6, *P* = 0.0008 for HER2 and ρ = 0.7, *P* = 0.015 for ER, Supplementary Figure 7C-D). The HER2-positive subtype had a statistically significantly higher average HER2 transcript absolute difference between adjacent tumor pseudo-cells, in comparison to Luminal B/HER2-positive tumors, indicating a higher level of spatial heterogeneity (13.3 *vs*. 8.7, Mann-Whitney test, *P* = 0.04, Supplementary Figure 7E). In contrast, no significant difference was observed for ER among different subtypes (Supplementary Figure 7F).

Lastly, we assessed how the difference in mRNA expression scales with the physical distance separating two cells, both using pseudo-cell and manually segmented cell data (Materials and Methods). In the case of HER2, we found that in most HER2-positive tumors there was a slight, but statistically significant positive correlation between local mRNA differences and physical distances among cells (Figure 5B and Supplementary Figure 8A-B). In contrast, ER-positive tumors comprised two groups: in some tumors, the local inter-cellular difference in ER transcript levels was positively correlated with the physical distance separating two cells, whereas in other tumors there was a weak, but statistically significant inverse correlation between the two metrics (Figure 5C). Overall, these data demonstrate that FFPE-smFISH is not only a robust diagnostic assay for assessing the HER2 and ER status, but that unlike standard HER2 and ER diagnostic methods, it can also be used to measure the spatial organization of these two key biomarkers within the tumor, yielding measures of spatial diversity that could in turn be correlated with clinical outcome.

## DISCUSSION

We have developed and validated a robust single-molecule RNA FISH protocol for the detection of both mature and nascent RNA in FFPE tissue sections (FFPE-smFISH), and applied it to quantify the expression and the intra-tumor spatial heterogeneity of two prominent breast cancer biomarkers, HER2 and ER. Single-molecule RNA visualization in FFPE tissue sections was previously achieved in prostate cancer biopsies [15] by combining the original single-molecule RNA FISH method [27] - which uses cDNA-derived probes labeled with multiple fluorophores instead of oligonucleotides with the use of sodium borohydride to reduce fixative-dependent autofluorescence, which is usually very high in clinical FFPE samples. However, this approach was shown to detect only native transcripts accumulated at the site of transcription in cells undergoing so-called transcriptional bursting [28], thus limiting a broader applicability. In contrast, the FFPE-smFISH protocol described here is able to detect both nascent as well as mature transcripts, and features the following improvements: 1) smFISH probes consist of single fluorescently labeled oligonucleotides [8] rather that cDNA labeled with multiple fluorophores (see also the scheme in Figure 1A), which provides more versatility and specificity and is easier to implement; 2) in addition to sodium borohydride, two RNA-retrieval steps are used to minimize autofluorescence (see the step-by-step FFPE-smFISH protocol in Supplementary Information), enabling the detection not only of bright transcription sites in the nucleus, but also of lower intensity signals corresponding to mature RNA. We note that although in this study we have produced HER2 and ER smFISH probes as described in the original smFISH method [8], more cost-effective and scalable probe production methods are now available (our unpublished data and [29, 30]). (These methods are designed to produce target-specific primary oligos that carry one or two genome-orthogonal sequence flap(s) to which a secondary fluorescently labeled oligo is hybridized).

A distinguishing feature of FFPE-smFISH over the methods that are routinely used for HER2 and ER diagnostics, such as IHC, DNA FISH, and chromogenic *in situ* hybridization (CISH), is that the signal generated by smFISH (i.e., fluorescent dots) is digital rather than analog. In IHC and CISH assays, the readout is a continuous or color spectrum, for which an arbitrary positivity threshold needs to be applied to discriminate signal from background. The fact that thresholding is very subjective challenges inter-laboratory and inter-operator reproducibility. In contrast, smFISH generates discrete, diffraction-limited fluorescence spots that can be robustly and automatically counted. Moreover, in contrast to DNA FISH which produces only a few signals per cell, in smFISH a few up to hundreds of individual RNA molecules per cell can be resolved, providing high sensitivity and statistical power (ultimately, the resolution depends on the number of transcripts and on the cell volume. For highly expressed genes, such as ribosomal RNA genes, it is not possible to resolve individual RNA molecules, but the total fluorescence intensity can be used as a proxy).

By imaging HER2 and ER transcripts in 49 archival breast cancer samples with HER2 and ER status previously established by IHC and DNA FISH, and by applying a simple and computationally efficient approach to segment the images in a regular grid of pseudo-cells, we demonstrate that FFPE-smFISH is able to accurately classify tumors belonging to different molecular subtypes, and has an excellent diagnostic performance (best AUC = 0.92 and 0.95 for HER2 and 0.86 and 0.90 for ER using pseudo-cells and manually segmented cells, respectively). Importantly, while the IHC and DNA FISH scores currently used in the clinic are categorical variables that provide limited information about inter-tumor heterogeneity, the FFPE-smFISH score (mRNA density per pseudo-cell) is a continuous variable that enables a more resolved patient stratification. Indeed, we observed that tumors in each IHC or DNA FISH group had considerable variability in HER2 and ER transcript levels. Improved stratification might be very useful in particular for the IHC 2+/DNA FISH-negative group, which is currently not eligible for anti-HER2 targeted therapy. We found that one tumor in this group had HER2 expression levels similar to tumors in the IHC 2+/DNA FISH-positive and IHC 3+ groups, suggesting that anti-HER2 therapy might have been applicable. Conversely, several tumors from patients in the IHC 2+/DNA FISH-positive group, who received targeted therapy in agreement with current guidelines [17], showed HER2 expression levels close to those in the IHC 2+/DNA FISH-negative group, raising the question whether some of these patients might have been overtreated (Supplementary Figure 2B). In the future, application of HER2 FFPE-smFISH to larger prospective cohorts of samples will be fundamental to clarify the clinical relevance of a more resolved IHC 2+ patient stratification.

Another important advantage of FFPE-smFISH over IHC or CISH is that, because DAPI-stained nuclei are imaged together with RNA molecules and their boundary is easy to segment, single-cell segmentation can be readily performed for large numbers of cells within the same tumor sample (in this study, we chose to manually segment over 38,000 cells in order to be maximally accurate in selecting only tumor cells. However, automatic segmentation of DAPI-stained nuclei is possible and technically less cumbersome and more accurate compared to cell segmentation in H&E or IHC images). This opens up a unique opportunity to quantify transcriptional heterogeneity at the single-cell level and at the same time explore the spatial organization of different cell populations in the tumor ecosystem. Indeed, by counting HER2 and ER transcripts in thousands of single-cells, we discovered that tumors belonging to the same subtype may harbor different population structures, with some tumors showing distinct transcriptionally ‘on’ and ‘off’ sub-populations. Notably, while in the case of ER the observed bimodality was also detected at the protein level after careful re-evaluation of the IHC images, in the case of HER2 the coexistence of ‘on’ and ‘off’ populations could not be detected at the protein level. This discrepancy might be explained by the fact that the IHC signal is not quantified on a continuous scale as the smFISH signal, or alternatively by the fact that, while smFISH quantification is done automatically on a large number of cells, IHC quantification is performed by eye on a limited number of cells. Alternatively, HER2 RNA levels might oscillate due to transcriptional bursting [28], while protein levels might be more stable. Furthermore, the ability to measure HER2 and ER transcripts simultaneously in the same cell gave us a unique opportunity to explore the crosstalk, at the transcriptional level, between the HER2 and ER pathways. We found no evidence of correlated HER2 and ER gene expression, except for two interesting cases which harbored distinct tumor cell sub-populations, including one with correlated HER2 and ER transcript levels. In the future, it will be important to extend these analyses to prospective patient cohorts in order to assess whether differences in the tumor population structure hold prognostic and/or predictive information.

Another key advantage of our approach is that, by performing quantitative measurements at multiple, distinct spatial locations in the tumor, we were able to measure various aspects of intra-tumor transcriptional heterogeneity, which might be very helpful to understand how the complex ecosystem of tumors influences therapy response and resistance. We used a mathematical tool originally developed in information theory - Shannon entropy - to characterize both the local and the global heterogeneity of HER2 and ER gene expression in all 49 samples in our cohort. The Shannon entropy metric has been widely used in ecology and was recently introduced in cancer research to measure genetic diversity in breast cancer [5–7] and to characterize the spatial heterogeneity of immune infiltrates in breast cancer [31]. We found that the diversity of HER2 and ER expression levels was higher locally in comparison to the whole tumor, but also that there was considerable intra- and inter-patient variability. Importantly, tumors belonging to the same molecular subtype had different transcriptional ITH profiles, suggesting that not only the type and average expression level, but also the spatial distribution of cells expressing a given biomarker might influence how a tumor evolves and responds to therapy. Along these lines, analysis of gene expression differences between adjacent cells - as demonstrated by our proof-of-principle analysis - could be very useful to model tumor evolution. We envision several forces that might be at work to shape the observed transcriptional variability among adjacent cells: 1) in one scenario, each tumor clone is relatively stable in its genetic and epigenetic state, however dispersive migration and physical mixing of different clones creates local heterogeneity (in this scenario, adjacent cells rarely originate from the same cell division, but rather come close to each other by active motion). 2) In the second scenario, high mutational rates (resulting, for instance, in rapid copy number changes or promoter mutations) or short-lasting epigenetic memory (resulting, for example, in a high frequency of transcriptional bursts) cause expression levels to rapidly diverge among adjacent cells. This in turn would generate diversity among clonal progenies that might increase the fitness of the tumor in the face of the constantly changing tumor microenvironment. In the future, it will be fascinating to test these hypotheses by combining FFPE-smFISH with other methods such as DNA FISH (we have previously shown that smFISH and high-resolution DNA FISH can be effectively combined in the same sample [23]) and STAR-FISH [7] to measure simultaneously copy number levels, selected mutations and gene expression in single cells, followed by mathematical modeling of the experimental data, for example by applying tools like the Chaste cell dynamics simulator (https://www.cs.ox.ac.uk/chaste). In particular, future applications of FFPE-smFISH and spatial analysis of gene expression in prospective patient cohorts will be extremely important to determine whether measuring transcriptional ITH is clinically useful.

Other methods have been used to detect RNA molecules *in situ* in FFPE tissue sections, including padlock probes and rolling circle amplification [11] and the branched FISH probes commercialized as RNAscope [9]. However, these methods typically require more steps and have a lower detection efficiency compared to smFISH. In contrast, the FFPE-smFISH protocol described here is a straightforward procedure with a relatively fast turnaround time (approx. 16-18 man-hours from deparaffinization to imaging). Importantly, fluorophore coupling of amino-modified oligos is easy to implement in any research laboratory, and does not require dedicated equipment. In the diagnostic setting, an important advantage of FFPE-smFISH over IHC is that the assay conditions are very robust and no *de novo* optimization is required for new probes. Moreover, FFPE-smFISH is particularly valuable for gene targets for which good antibodies are currently unavailable or when antibody staining is not sufficiently sensitive. One limitation is that, especially in FFPE tissue sections with high autofluorescence, a 100X magnification lens is needed to detect a clear signal, therefore limiting the portion of the tumor that can be imaged in a relatively short time. However, our study shows that 30-50 fields of view sparse throughout a single tumor section (which can be imaged in a fully automated manner in approx. 2 hours) are completely sufficient to perform a reliable determination of the HER2 and ER status, and to quantify transcriptional ITH. In conclusion, FFPE-smFISH is a versatile, easy-to-implement and robust method, which can find numerous applications in diagnostics, and paves the way for studies aimed at assessing the clinical impact of intra-tumor transcriptional heterogeneity.

## MATERIALS AND METHODS

### Samples

FFPE samples from 49 patients diagnosed with breast cancer between 2010 and 2013 were retrieved at the Breast Unit of ‘Azienda Ospedaliera Universitaria Città della Salute e della Scienza di Torino’, University of Turin, Italy. The study was conducted under ethical permission granted by the Committee for human Biospecimen Utilization (DSM-ChBU) of the Department of Medical Sciences, University of Turin, Italy. Written informed consent was obtained from all cancer patients for collection, storage and research use of both fresh and archival tumor samples. Clinical and pathological information is summarized in Table 1 and 2 and in Supplementary Table 3.

### Immunohistochemistry and DNA FISH

IHC data for estrogen receptor (ER), progesterone receptor (PR), and Ki67 were retrieved from the original pathology reports. The following antibodies were used for IHC: for HER2, the HercepTest^TM^ Kit (Dako, Cat. K5207); for ER, the rabbit mAb, clone SP1 (Ventana-Diapath, Tucson, AZ); for PR the rabbit mAb, clone 1E2 (Ventana-Diapath). The proliferation index was assessed using the Ki67 mouse mAb (clone MIB-1, Dako). Thresholds for positivity were according to the 2013 ASCO/CAP guidelines [17]: ≥ 1% for ER, > 20% for PR, score 3+ or score 2+/HER2-amplified for HER2 and > 14% for Ki67. In all the samples we also performed DNA FISH using probes for HER2 and CEP17 (Abbott Laboratories, PathVysion HER-2 DNA Probe Kit II CE, Cat. 06N46-035). For analysis, 10 invasive areas on each slide were selected and automatically acquired at 40X magnification with the motorized Metafer scanning system (Zeiss) and the Axio Imager epifluorescence microscope (Zeiss). The PathVysion V2 software was used to analyze the results. DNA FISH data were scored according to the 2013 ASCO/CAP guidelines [17]. HER2 positivity was defined as IHC score 3+ or IHC score 2+ with HER2 amplification. For molecular subtypes, we used the IHC-based surrogate classification proposed by the St. Gallen International Expert Consensus, which includes five categories: Luminal A (ER-positive/PR-positive/HER2-negative/Ki67-low); Luminal B/HER2-negative; Luminal B/HER2-positive (ER-positive/HER2-positive); HER2-positive; and triple-negative (ER-negative/PR-negative/HER2-negative) [32]. The Luminal B/HER2-negative category included ER-positive carcinomas with Ki67 > 14% [33] and/or PR < 20% [34].

### smFISH

Probes targeting HER2 and ER were designed based on our previously described database covering all human transcripts (www.fusefish.eu [13]). Probes consisted of the oligonucleotides listed in Supplementary Table 1. We purchased oligos with a 3′-TEG amino modification from Biosearch Technologies, and coupled them to either Cy™5 (GE Healthcare, cat. Q15108) or Alexa Fluor^®^ 594 (Molecular Probes, cat. A20004). A step-by-step protocol is available in Supplementary Information. Briefly, 3 μm-thick FFPE tumor block sections were mounted on coverglasses coated with poly-L-lysine (Sigma). After deparaffinization in xylene, tissue sections were post-fixed for 5 min in methanol-acetic acid 3:1 (v/v), rehydrated, and then heated for 45 min at 80°C in 0.01 M sodium citrate pH 6 supplemented with ribonucleoside vanadyl complex (RVC, NEB, cat. S1402S) diluted 1:20 (v/v). All deparaffinization steps were performed in special plastic jars (EMS, cat. 71385) that had been thoroughly decontaminated with RNaseZap^®^ (Ambion, cat. AM9780). After dehydration, 22×22 mm “Secure Seal” hybridization chambers (EMS, cat. 70333-10) were mounted on each coverglass, covering as much tumor tissue as possible. Tissues were rehydrated and treated for 15 min with 0.025% pepsin in 10 mM HCl. Auto-fluorescence was reduced by repeatedly flushing the chamber with freshly prepared 1% NaBH_4_ in 1X PBS solution, over a period of 15 min at room temperature. After washing in RNase-free water, samples were stored in 2X SSC buffer (Ambion, cat. 9763) at 4°C until hybridization was performed. Samples were hybridized as previously described [13]. All solutions were prepared in RNase-free water (Ambion, cat. AM9939).

### Image acquisition

We imaged all 49 cases at 100X magnification on an inverted epifluorescence microscope (Nikon) equipped with a high-resolution CCD camera (Pixis, Princeton Instruments) controlled by MetaMorph. Per region of interest, we acquired an image stack consisting of 5 focal planes spaced 0.4 μm apart.

For the proof-of-principle visualization of smFISH dots on top of a scan of the same tissue section stained with H&E presented in the supporting website http://tumorheterogeneity.eu/ we used a custom-designed inverted epifluorescence microscope (Eclipse Ti-E, Nikon) equipped with an EMCCD camera (iXON Ultra 888, Andor) controlled by NIS Elements software (Nikon). First, we imaged smFISH signals at 100X magnification in selected tumor regions. For each region of interest, we acquired an image stack consisting of 5 focal planes spaced 0.3 μm apart. Afterwards, we washed the tissue section, stained it with Hoechst 33342, and scanned it with a 40X magnification objective. The size of the tissue scan was 1 × 1 cm. Lastly we stained the same tissue section with H&E and scanned it using a 10X magnification objective.

### smFISH signal quantification

In each field of view, we identified smFISH signals corresponding to individual mRNA molecules using custom-made scripts in MATLAB^®^, as previously described [13]. For the analysis based on pseudo-cells, we split each image into a regular grid of squares (i.e., pseudo-cells). We compared seven grids (9 × 9, 10 × 10, 11 × 11, 12 × 12, 13 × 13, 14 × 14, and 16 × 16 pseudo-cells) for which the pseudo-cell area would fall in the inter-quartile range of the area of manually segmented cells at six different expansion margin lengths (see Supplementary Figure 1B). For each pseudo-cell, we calculated the mRNA density (dots/μm^3^) by dividing the total number of mRNA spots in the pseudo-cell by the number of focal planes minus one times the distance between each plane times the pseudo-cell area. In order to account for possible background dots, we compared four different thresholds of the number of transcripts per pseudo-cell (≥ 0, 1, 2, or 3 transcripts).

For the analysis based on single-cell segmentation, we first manually segmented the edge of tumor cell nuclei stained with DAPI using segmentation polygons of a variable number of edges. In order to define an approximate cell boundary, we uniformly dilated the segmentation polygons by 0, 5, 15, 20, or 25 pixels (1 pixel = 125 nm). Since the cells were not imaged throughout their full thickness in the z direction, we computed mRNA densities by dividing single-cell mRNA counts by the volume of the prism with base corresponding to the segmentation polygon, and height equal to 0.4 μm multiplied by the number of image planes minus one. All data analyses were performed in MATLAB^®^ using custom-made scripts.

### Automatic identification of regions containing cells

To count smFISH dots only in image regions containing cells, we first quantified the local structure variations of the z-projection of each DAPI image, assuming that the variation is higher inside nuclei in comparison to the rest of the image. To this end, we calculated the gradient structure tensor [35] using σ gradient = 250 nm and σ tensor = 875 nm and manually set a threshold, common to all images, on the determinant of the structure tensor. We then computed the density of smFISH dots within the identified nuclei-containing regions, and compared the results with the smFISH score obtained using pseudo-cells.

### Registration of smFISH dots on hematoxylin-eosin tissue scans

First, we used the DAPI channel to register each 100X image onto the large scan of the same tissue section consisting of multiple 40X images stitched together. In order to match the resolution of the stitched 40X image, we first downscaled all the 100X images. We then applied normalized cross-correlation to find the best location of each 100X image within the 40X stitched image, capturing the scaling and translation in the affine transformation matrix, A1. Next, we downscaled the 40X stitched DAPI image to match the size of the 10X stitched H&E image. By manually selecting a few reference points in each image, we set up a transformation matrix, A2, mapping between the images. Lastly, we loaded the smFISH dots, D from each 100X image and converted their coordinates into the coordinate system of the H&E image by the transformation: D’ = A2 A1 D. All these operations were run in MATLAB^®^ using custom-made scripts. To interactively visualize the smFISH dots overlaid onto the H&E images, we created deepzoom images (http://search.cpan.org/~drrho/Graphics-DZI-0.05/script/deepzoom) and displayed them using OpenSeadragon (https://openseadragon.github.io/).

### Receiver operating characteristic (ROC) analysis

We set 200 arbitrary cutoff values for calling the smFISH score positive or negative, by multiplying each value in the integer interval [1,200] by 0.001. For each cutoff value, *t* we calculated the number of true positive (TP), false positive (FP), true negative (TN) and false negative (FN) cases as follows:

TP = (smFISH score ≥ *t*) AND (reference test is positive)

FP = (smFISH score ≥ *t*) AND (reference test is negative)

TN = (smFISH score < *t*) AND (reference test is negative)

FN = (smFISH score < *t*) AND (reference test is positive)

where the reference test was IHC and DNA FISH for HER2, and IHC for ER (as described above, HER2 positivity was defined as IHC score 3+ or IHC score 2+ with HER2 amplification, whereas ER positivity was defined as ≥ 1% of positive tumor cells). For each cutoff value, we then calculated the sensitivity and the specificity as follows:

sensitivity = TP / (TP + FN)

specificity = TN / (TN + FP)

Lastly, for each cutoff value we plotted the corresponding (1 - specificity) value on the x-axis and the sensitivity on the y-axis (ROC curve). For both HER2 and ER, we computed the maximum specificity and sensitivity (i.e., the (x,y) pair closest to the top left corner of the graph), as well as the area comprised between the ROC curve and the plot diagonal (AUC). All the calculations were run using a custom-made script written in MATLAB^®^. We separately calculated the AUC for different pseudo-cell sizes and thresholds of dots per pseudo-cell (for pseudo-cells) as well as for different expansion lengths of the segmentation polygons (for manually segmented cells), and then plotted as heatmap the resulting vectors or matrices.

### RNA extraction and real-time PCR

From each of the selected FFPE tumor blocks, we cut five consecutive 10 μm-thick sections and collected them in a 1.5 ml RNAse-free Eppendorf tube. We performed RNA isolation using the MasterPure™ Purification kit (Epicentre, cat. MC85200). We deparaffinized the sections by incubations in xylene followed by incubations in 100% ethanol. After the washes in ethanol, we air-dried the pellet for several minutes at room temperature before Proteinase K treatment, according to the “Method 3” for FFPE tissues described in [36]. We resuspended the RNA pellet in nuclease-free water, and measured the RNA concentration with a NanoDrop™ Spectrophotometer (Thermo Fisher Scientific). We performed a DNase treatment step with the TURBO DNA-free^TM^ Kit (Ambion, cat. 1907), after which we reverse transcribed a total of 4 μg of RNA to cDNA using the High-Capacity cDNA Reverse Transcription Kit (Thermo Fisher Scientific, cat. 4368814). As negative controls for DNA contamination, we repeated the same procedure skipping the reverse transcriptase. We amplified the obtained cDNA by real-time PCR using the Power SYBR^®^ Green PCR Master Mix and the StepOne machine (Applied Biosystems), according to the manufacturer's protocol. The following primers (final concentration 50 nM) were used to perform real-time PCR:

ACTB forward: 5′-CTCACCATGGATGATGATATCGC

ACTB reverse: 5′-AGGAATCCTTCTGACCCATGC

ERBB2 forward: 5′-GTGTGGACCTGGATGACAAGGG

ERBB2 reverse: 5′-GCTCCACCAGCTCCGTTTCCTG

For each primer pair, we included one no-template control. We carried out the real-time PCR reaction as following: 10 min incubation at 95°C followed by 40 cycles at 95°C for 15 s and 60°C for 1 min for annealing and elongation. To reduce the risk of contamination from previously amplified products, separate areas were used for RNA isolation and PCR. We used ACTB as a reference gene to normalize the gene expression data of ERBB2 between the samples. We applied the 2^−ΔΔCt^ method to determine and analyze the relative changes in ERBB2 expression between the different samples.

### Multiplex ligation-dependent probe amplification (MLPA)

We dewaxed two 4 μm-thick paraffin sections and mesodissected them manually as follows: we scraped off tumor areas with a pipet tip and collected them into a DNase-free tube, as previously reported [37] (infiltrating carcinoma were recognized by comparison with a serial H&E stained slide, and areas of ductal carcinoma *in situ* were discarded). We extracted genomic DNA using the PureLink^®^ Genomic DNA kit (Thermo Fisher Scientific, cat. K182001) following manufacturers’ instructions. We performed MLPA reactions with 100-200 ng of purified genomic DNA using the P004-C1 ERBB2 probe mix (MRC-Holland) and a MJ Thermalcycler (MJ Research). We separated the PCR products on an ABI 3130 capillary sequencer (Applied Biosystems) and analyzed gene copy numbers using GeneMapper 4.0 (Applied Biosystems) and Coffalyser (version 8.0 MRC-HOLLAND) software. Because four probes targeting HER2 are included in the kit (17-035.1 ERBB2 exon 07, 17-035.1 ERBB2 exon 22, 17-035.1 ERBB2 exon 28, 17-035.1 ERBB2 exon 29), we calculated the mean of each probe-specific normalized ratio of the HER2 gene. A mean value below 1.3 was defined as normal, between 1.3 and 2.0 as gain and above 2.0 as high level amplification, as previously reported [38, 39].

### *In situ* proximity ligation assay (PLA)

We performed HER2 PLA using the Duolink II detection kit (O-Link Bioscience) according to the manufacturer's instructions. As primary antibody directed against HER2 we used the Polyclonal Rabbit Anti-Human c-erbB-2 (Dako, cat. A0485). We imaged the samples using the Metafer Scanning System and AxioImager epifluorescence microscope equipped with a 40X objective. We consistently acquired an image stack consisting of 9 focal planes spaced 0.3 μm apart per region of interest (in total, 10 regions of interest per case). We analyzed the data using the Duolink Image Tool.

### Statistical comparisons

For all comparisons, we determined the statistical significance using the Mann-Whitney test (two-tailed). For correlation analyses, we calculated the non-parametric Spearman correlation coefficient and performed linear regression whenever shown. All statistical analyses were done either using GraphPad Prism or MATLAB^®^.

### Intra-tumor heterogeneity analysis

The local diversity index was calculated using pseudo-cell data as follows: for each image, *i* in a given case, we first computed how many different expression levels, *L*i (i.e., the number of smFISH dots per pseudo-cell) were detected (for HER2 we considered only pseudo-cells with ≥ 3 dots, whereas for ER only pseudo-cells with ≥ 1 dot). We then calculated the probability, *p*il defined as the fraction of pseudo-cells in the image, *i* with expression level, *l*i. Lastly, for each image, we calculated the Shannon diversity index as:

$$H_{i}=-\sum_{l=1}^{L_{i}}p_{i,l}\;\log_{2}\;p_{i}.$$

In order to compare different images within the same tumor as well as different tumors, we normalized *H*i as:

$$\bar{H_{i}}=\frac{H_{i}}{\log_{2}\;L_{i}}$$

This normalized value was defined as our local diversity index. The global diversity index was obtained in a similar way, except that expression levels were calculated by pooling together all pseudo-cells from different fields of view. Local and global diversity indexes for either HER2 or ER were only computed for tumors previously scored as positive by IHC and/or DNA FISH.

To assess the spatial variation of HER2 and ER transcripts based on pseudo-cells, we first calculated the difference of mRNA counts between each pseudo-cell in a field of view and each of its 8 neighbors (for HER2 we considered only pseudo-cells with ≥ 3 dots, whereas for ER only pseudo-cells with ≥ 1 dot). Additionally, we computed all the pairwise Euclidean distances as well as the absolute difference of transcript counts between all the thresholded pseudo-cells in a given field of view. As a result, for each tumor we obtained one vector of inter-pseudo-cell physical distances and one vector of expression differences. To assess whether the two vectors were correlated, we calculated the Spearman correlation coefficient and the *P* value for its statistical significance. The same type of analysis was done using manually segmented cells, with the exception that in this case we calculated the distance and expression difference between each segmented cell in a given field of view and the other segmented cells in the same image. We only analyzed tumors that had been scored as positive by IHC and/or DNA FISH. All data analyses were performed in MATLAB^®^ using custom-made scripts.

## SUPPLEMENTARY MATERIALS FIGURES AND TABLES






