# Supplementary material for: Quantification of HER2 and estrogen receptor heterogeneity in breast cancer by single-molecule RNA fluorescence in situ hybridization
Source: Oncotarget. 2017 Feb 25;8(12):18680–98. doi: 10.18632/oncotarget.15727 (PMC5386639; doi:10.18632/oncotarget.15727)
Supplement: Supplementary file 1 [file oncotarget-08-18680-s001.pdf]

Quantification of HER2 and estrogen receptor heterogeneity in breast cancer by single-molecule RNA fluorescence *in situ* hybridization

Supplementary Material

Supplementary Table 1. Due to its large size, this table is provided as a Supplementary Excel file.

Supplementary Table 2. Summary statistics of regions imaged and cells segmented in each case.

Molecular Subtype	Mean images/case	Segmented cases	Mean segmented images/case	Mean segmented cells/case
LumA	60	9	59	1210
LumB/HER2-neg	64	9	59	1362
LumB/HER2-pos	61	11	56	716
Triple-neg	55	2	53	490
HER2	59	5	55	1236

Supplementary Table 3. Due to its large size, this table is provided as a Supplementary Excel file.

For Supplementary Tables 1 and 3, see separate Supplementary Files

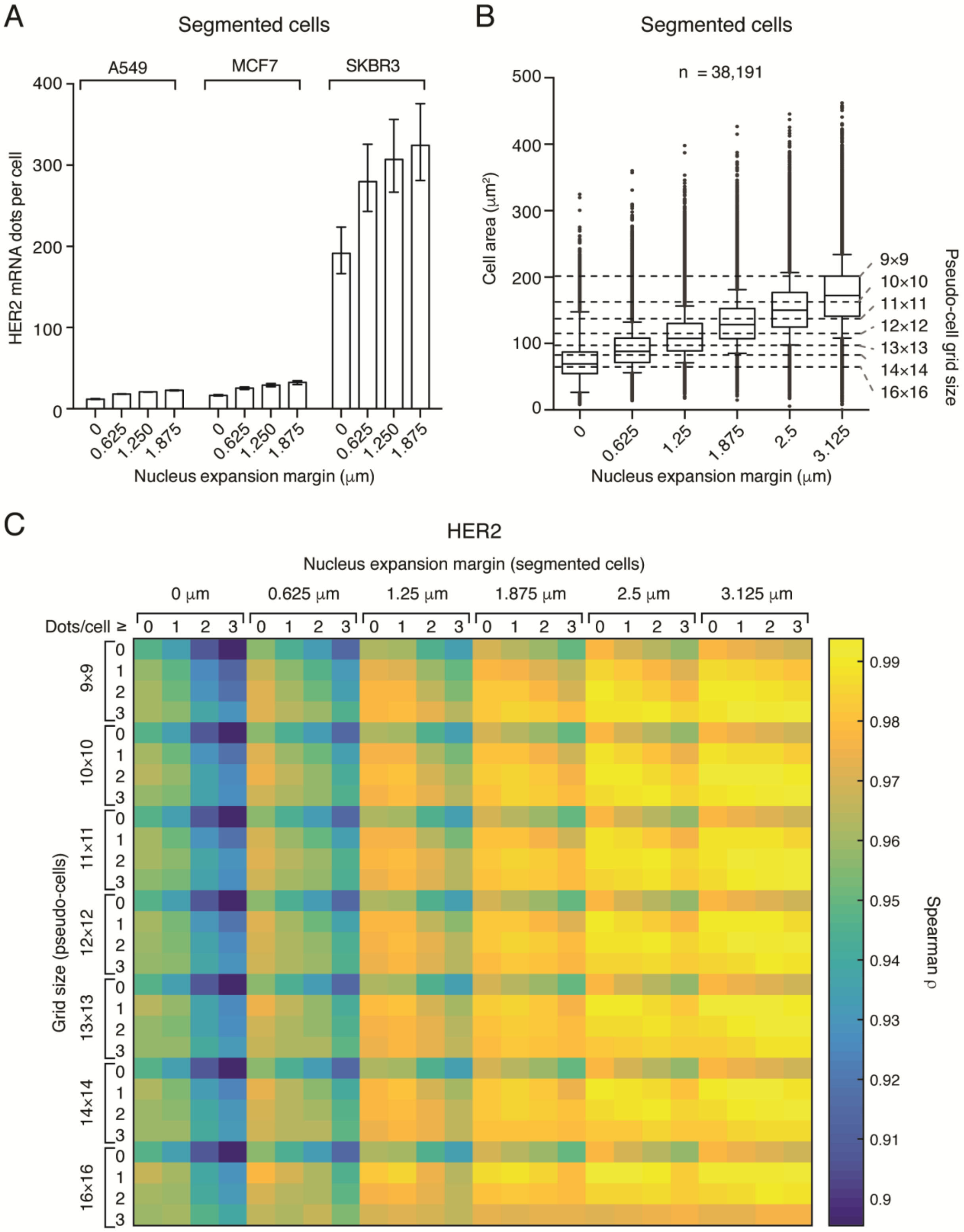
A
Segmented cells
A549
MCF7
SKBR3
HER2 mRNA dots per cell
0
100
200
300
400
0
0.625
1.250
1.875
Nucleus expansion margin (μm)
B
Segmented cells
n = 38,191
Cell area (μm²)
0
100
200
300
400
500
0
0.625
1.25
1.875
2.5
3.125
Nucleus expansion margin (μm)
9×9
10×10
11×11
12×12
13×13
14×14
16×16
Pseudo-cell grid size
C
HER2
Nucleus expansion margin (segmented cells)
0 μm
0.625 μm
1.25 μm
1.875 μm
2.5 μm
3.125 μm
Dots/cell ≥
0
1
2
3
Grid size (pseudo-cells)
9×9
10×10
11×11
12×12
13×13
14×14
16×16
0.99
0.98
0.97
0.96
0.95
0.94
0.93
0.92
0.91
0.9
Spearman ρ

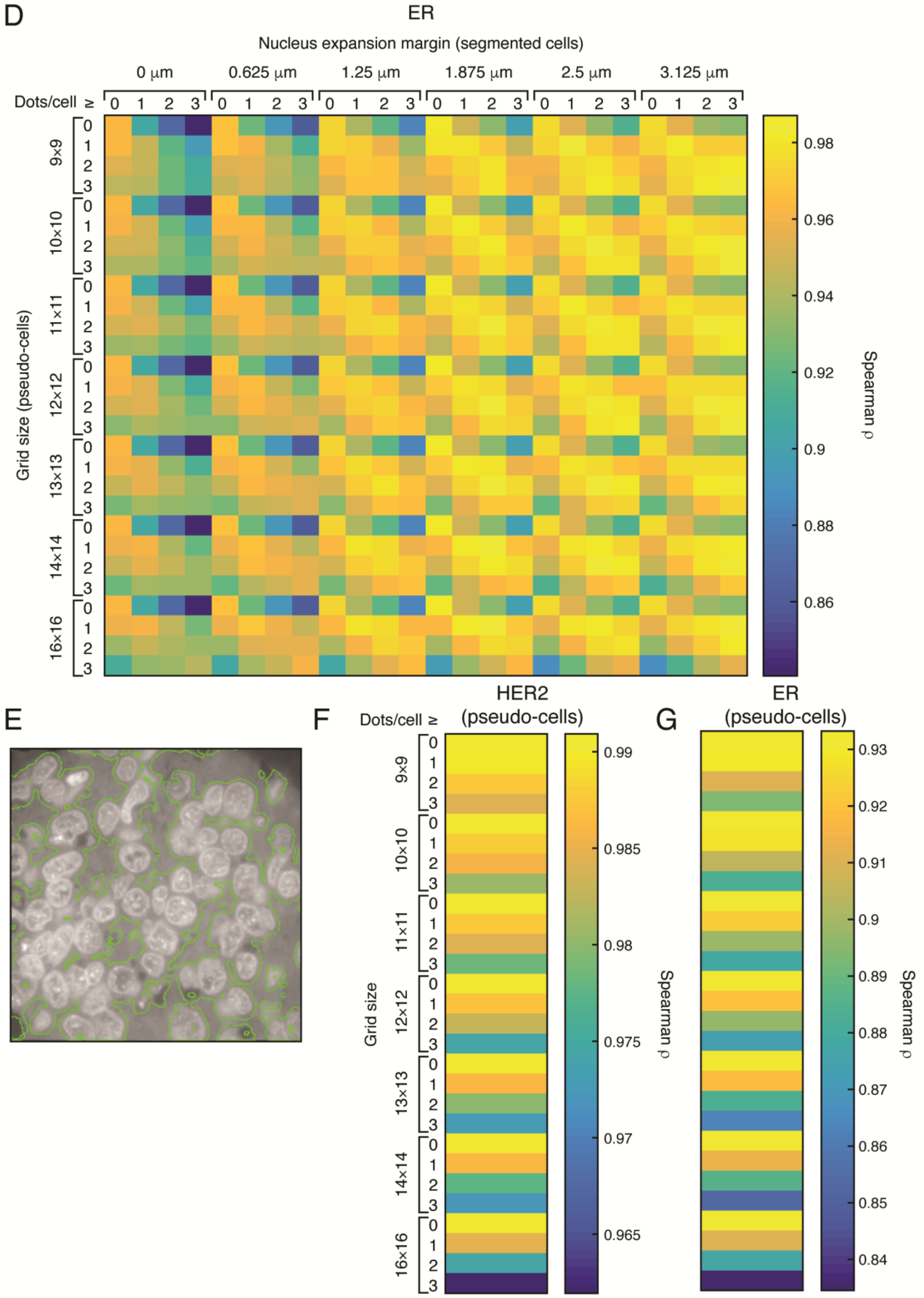
D
ER
Nucleus expansion margin (segmented cells)
0 μm
0.625 μm
1.25 μm
1.875 μm
2.5 μm
3.125 μm
Dots/cell ≥
Grid size (pseudo-cells)
9×9
10×10
11×11
12×12
13×13
14×14
16×16
Spearman ρ
0.98
0.96
0.94
0.92
0.9
0.88
0.86
E
F
HER2
(pseudo-cells)
Grid size
0.99
0.985
0.98
0.975
0.97
0.965
G
ER
(pseudo-cells)
0.93
0.92
0.91
0.9
0.89
0.88
0.87
0.86
0.85
0.84

Supplementary Figure 1. Comparison of the pseudo-cell *vs.* single-cell segmentation approach for FFPE-smFISH signal quantification. (**A**) Reproducibility of the single-cell segmentation approach for counting HER2 mRNA dots per cell, in three different cell lines (A549, MCF7, SKBR3). In order to include the mRNAs located in the cytoplasm, different expansion margins were used to dilate the boundary of nuclei stained with DAPI (see **Materials and Methods**). Bars: mean values. Whiskers: min-max range. (**B**) Distribution of the area of 38,191 cells manually segmented in 36 out of the 49 cases analyzed, obtained at increasing dilation margins of the boundary of nuclei stained with DAPI. The dashed lines indicate the area of that each pseudo-cell would have, if a pseudo-cell segmentation approach and grid sizes listed on the right were used instead. As it can be seen, these pseudo-cell areas fall in the range of most frequent segmented cell area values obtained at different expansion margins. (**C**) Correlation matrix of the HER2 transcript density in pseudo-cells *vs.* manually segmented cells, for different pseudo-cell sizes, nucleus expansion margins, and thresholds of dots per (pseudo-)cell. (**D**) Same as in (**C**), but for ER. (**E**) Identification of cell-containing regions inside an image based on the spatial pattern of DAPI intensity (see **Materials and Methods**). The green lines mark the boundary between cell-containing and cell-free regions. (**F**) Correlation matrix of the mean mRNA density in pseudo-cells *vs.* the mean mRNA density calculated in all the cell-containing regions identified as shown in (**E**), for different pseudo-cell sizes and thresholds of the number of smFISH dots per pseudo-cell. (**G**) Same as in (**F**), but for ER.

Supplementary Figure 2

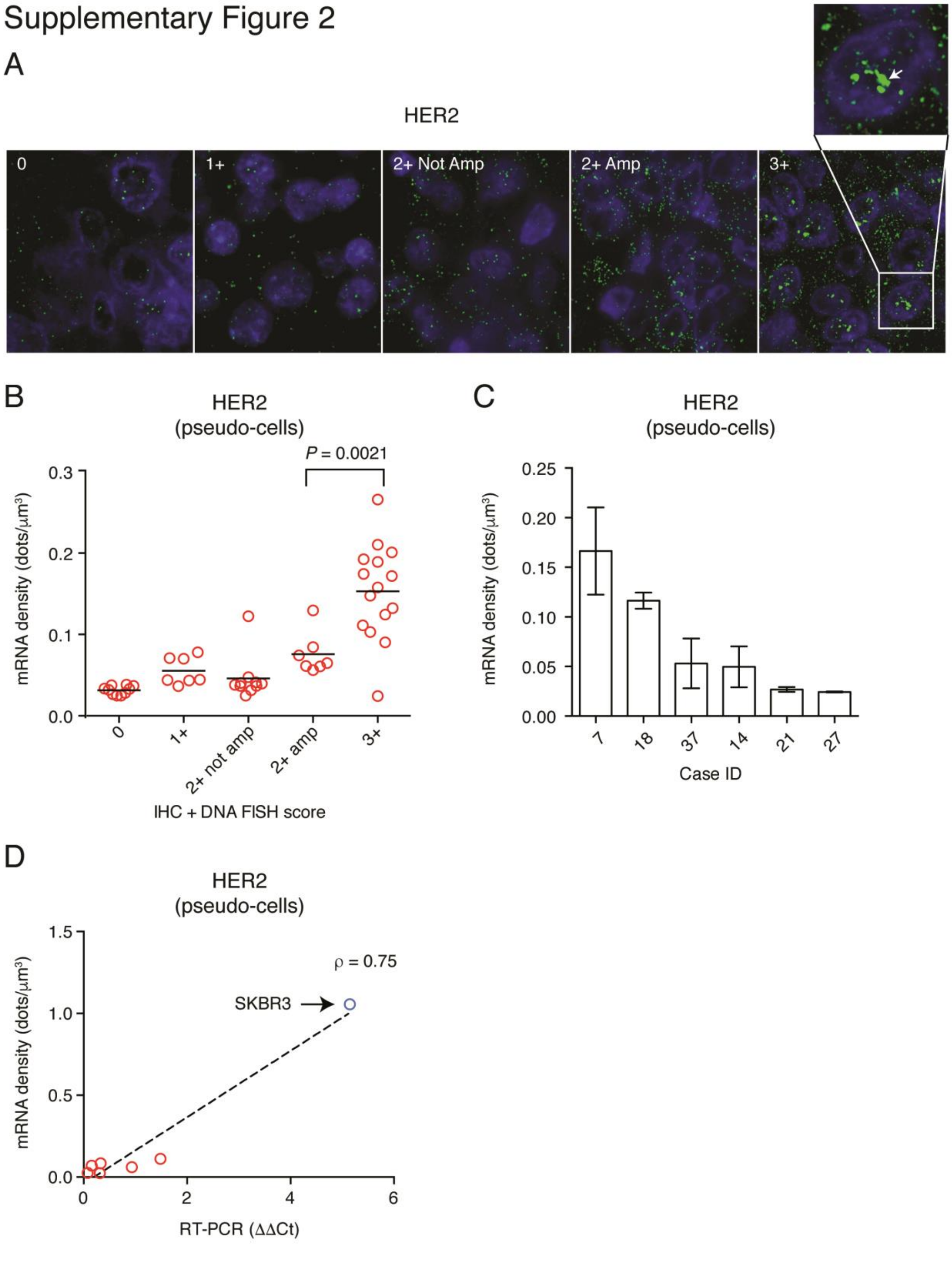

Supplementary Figure 2. Accuracy and reproducibility of HER2 scoring based on pseudo-cells. (**A**) Increasing HER2 mRNA abundance (green dots) in samples with increasing IHC HER2 score. Amp (amplified) and Not Amp (not amplified) refer to the HER2 status assessed by DNA FISH. The white arrow in the magnified inset indicates the accumulation of nascent HER2 mRNAs at the site of the amplified HER2 gene locus. (**B**) Mean HER2 transcript density per pseudo-cell in different tumors grouped based on HER2 IHC and DNA FISH. Amp (amplified) and Not Amp (not amplified) refer to the HER2 status assessed by DNA FISH. Each dot corresponds to the mean pseudo-cell transcript density in one of the 49 analyzed cases. Horizontal black bars represent the mean of all cases in the corresponding group. (**C**) Reproducibility of the mean HER2 transcript density per pseudo-cell calculated from two replicate FFPE experiments in 6 of the 49 cases analyzed. Bars: mean value. Whiskers: min-max range. (**D**) HER2 expression levels assessed by FFPE-smFISH *vs.* reverse transcription real-time PCR (RT-PCR) in 6 of the 49 cases analyzed. As comparison, we also quantified HER2 mRNA in SKBR3 cells, confirming by both smFISH and RT-PCR that this immortalized cell line expresses much higher levels of HER2 in comparison to primary tumor samples. Each red dot corresponds to one of the 6 cases analyzed. ρ, Spearman correlation coefficient.

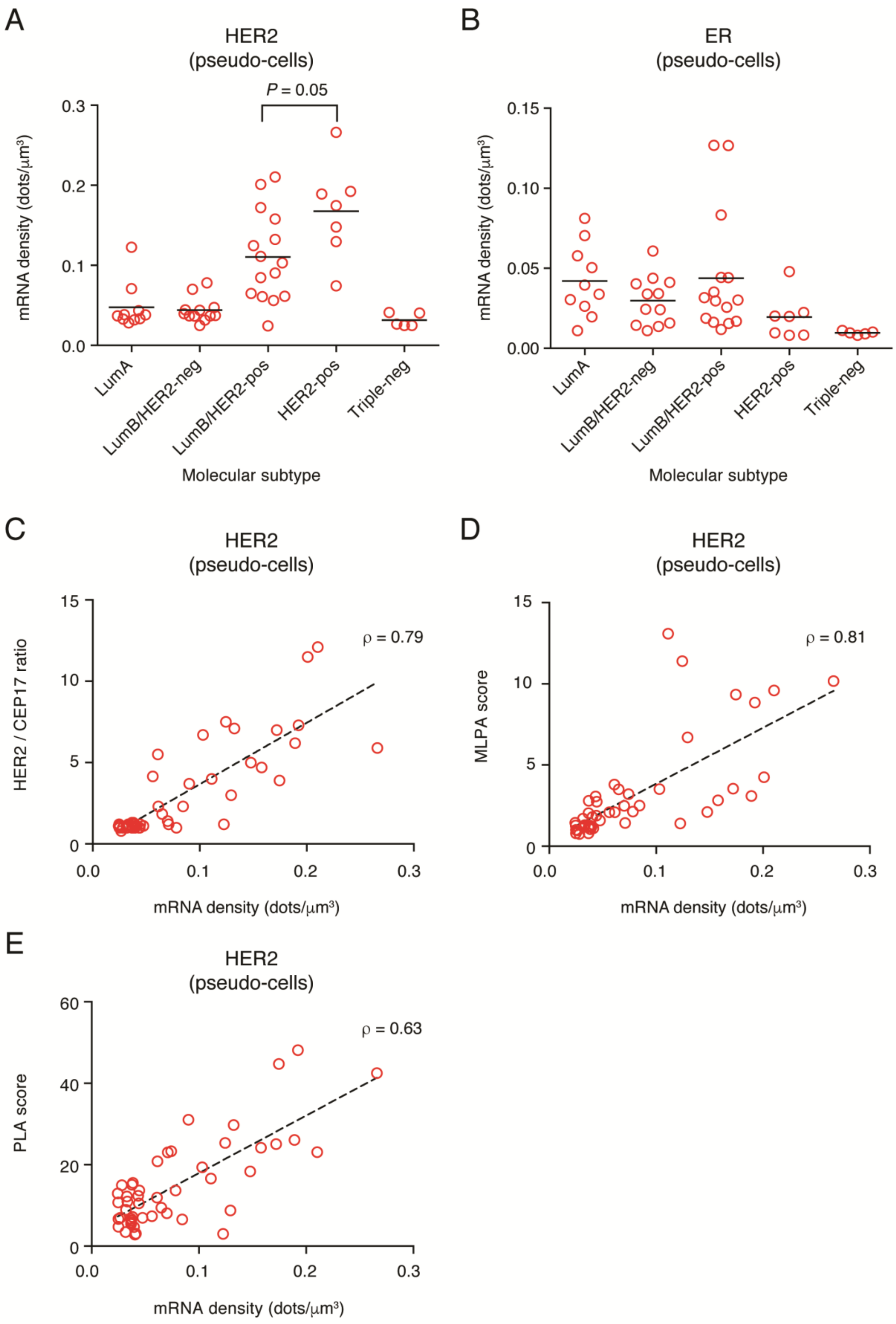
A
HER2
(pseudo-cells)
P = 0.05
mRNA density (dots/µm³)
0.3
0.2
0.1
0.0
LumA
LumB/HER2-neg
LumB/HER2-pos
HER2-pos
Triple-neg
Molecular subtype
B
ER
(pseudo-cells)
mRNA density (dots/µm³)
0.15
0.10
0.05
0.00
LumA
LumB/HER2-neg
LumB/HER2-pos
HER2-pos
Triple-neg
Molecular subtype
C
HER2
(pseudo-cells)
ρ = 0.79
HER2 / CEP17 ratio
15
10
5
0
0.0
0.1
0.2
0.3
mRNA density (dots/µm³)
D
HER2
(pseudo-cells)
ρ = 0.81
MLPA score
15
10
5
0
0.0
0.1
0.2
0.3
mRNA density (dots/µm³)
E
HER2
(pseudo-cells)
ρ = 0.63
PLA score
60
40
20
0
0.0
0.1
0.2
0.3
mRNA density (dots/µm³)

Supplementary Figure 3. Validation of FFPE-smFISH results obtained using the pseudo-cell segmentation method. (**A**) Mean HER2 transcript density per pseudo-cell in different molecular subtypes defined according to the St. Gallen surrogate intrinsic subtype classification (see **Materials and Methods**). Each dot corresponds to the mean pseudo-cell transcript density in one of the 49 analyzed cases. (**B**) Same as in (**A**), but for ER. (**C**) Correlation between the mean HER2 transcript density per pseudo-cell and the DNA FISH HER2/CEP17 ratio obtained for the same sample. Each dot corresponds to the mean pseudo-cell transcript density in one of the 49 analyzed cases. ρ, Spearman correlation coefficient. (**D**) Correlation between the mean HER2 transcript density per pseudo-cell and the HER2 score obtained by Multiplex Ligation-dependent Probe Amplification (MLPA) using genomic DNA extracted from the same sample. Each dot corresponds to the mean pseudo-cell transcript density in one of the 49 analyzed cases. ρ, Spearman correlation coefficient. (**E**) Correlation between the mean HER2 transcript density per pseudo-cell and the HER2 score obtained by Proximity Ligation Assay (PLA) performed in a separate tissue section from the same sample. Each dot corresponds to the mean pseudo-cell transcript density in one of the 49 analyzed cases. ρ, Spearman correlation coefficient.

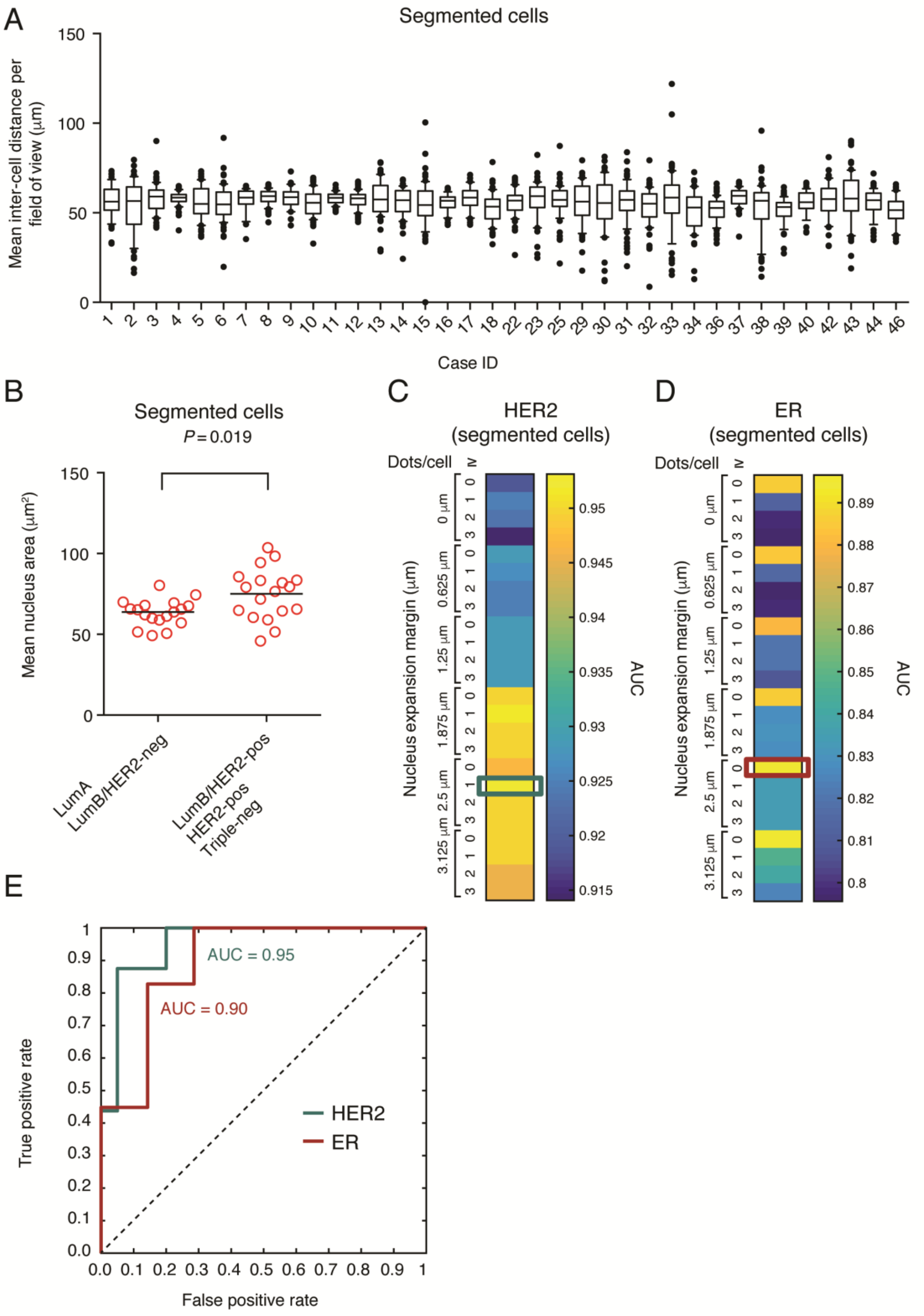
A
Segmented cells
Mean inter-cell distance per field of view (µm)
Case ID
B
Segmented cells
P = 0.019
Mean nucleus area (µm²)
LumA
LumB/HER2-neg
LumB/HER2-pos
HER2-pos
Triple-neg
C
HER2
(segmented cells)
Dots/cell ≥
Nucleus expansion margin (µm)
AUC
D
ER
(segmented cells)
E
True positive rate
False positive rate
AUC = 0.95
AUC = 0.90
HER2
ER

Supplementary Figure 4. Implementation and diagnostic accuracy of HER2 and ER scoring based on the single-cell segmentation approach. (**A**) Distribution of the mean distance between all segmented cells in the same field of view in the 36 manually segmented cases. Whiskers, 10–90 percentile. Case IDs refer to the list in **Supplementary Table 3**. (**B**) Mean area of segmented nuclei in less aggressive (Luminal A and Luminal B/HER2-negative) and more aggressive molecular subtypes (HER2-positive, Luminal B/HER2-positive and triple-negative). Each dot corresponds to the mean nucleus area in one of the 36 manually segmented cases. The *P* value was obtained with the Mann-Whitney test (two-tailed). (**C**) Heatmap showing different values of the HER2 area under the curve (AUC) obtained based on the ROC curve method for different expansion margins of the boundary of nuclei stained with DAPI and thresholds of the number of mRNA dots per cell (see **Materials and Methods**). Cyan box, nucleus expansion margin and threshold combination yielding the HER2 ROC curve shown in (**E**), and used in all subsequent single-cell analyses. (**D**) Same as in (**C**), but for ER. Red box, nucleus expansion margin and threshold combination yielding the ER ROC curve shown in (**E**), and used in all subsequent single-cell analyses. (**E**) Diagnostic performance of HER2 and ER FFPE-smFISH, obtained with the nucleus expansion margin and threshold combinations indicated by the cyan and red boxes in (**C**) and (**D**), respectively.

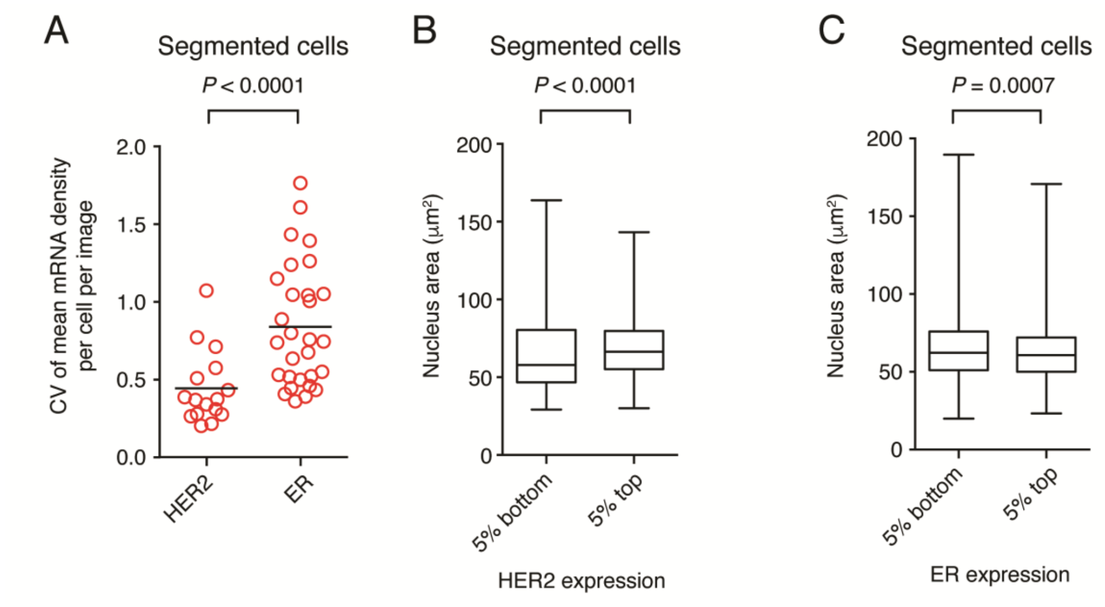

Supplementary Figure 5. Inter-regional variation of HER2 and ER gene expression in single cells. (**A**) Coefficient of variation (CV) of HER2 and ER mean mRNA density per segmented cell calculated for each field of view in HER2-positive and ER-positive cases, respectively. Each dot represents the CV of the vector of mean mRNA density per cell per image values obtained for each case. Horizontal black bars represent the mean of all cases in the corresponding group. The *P* value was obtained with the Mann-Whitney test (two-tailed). (**B**) Distribution of the area of segmented nuclei for cells in the upper and bottom 5th percentile of HER2 expression in HER2-positive and Luminal B/HER2-positive molecular subtypes. (**D**) Distribution of the area of segmented nuclei for cells in the upper and bottom 5th percentile of ER expression in Luminal A and B molecular subtypes. Whiskers, min–max range. The *P* values were obtained with the Mann-Whitney test (two-tailed).

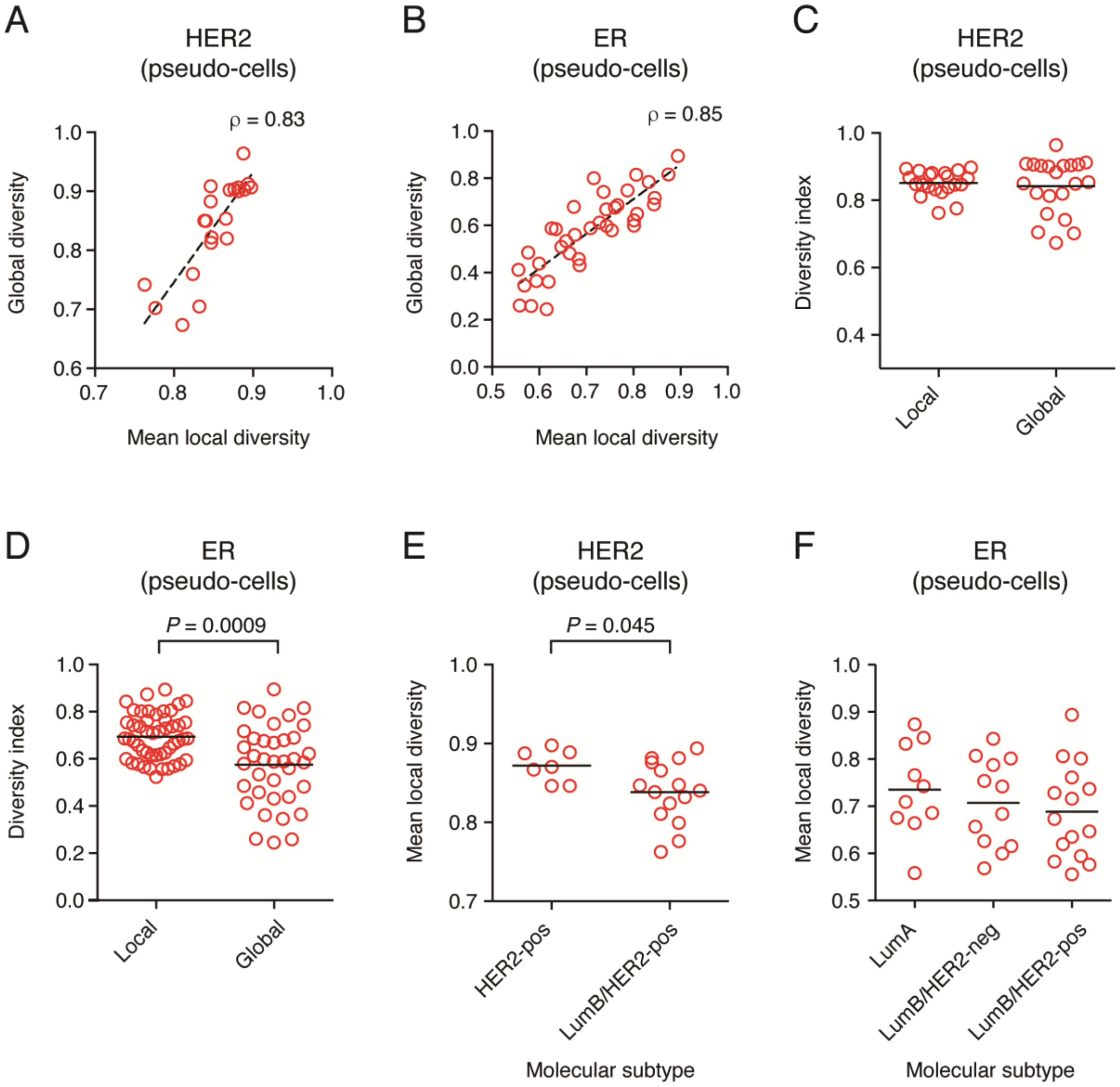

Supplementary Figure 6. (**A**) Spearman correlation between the mean normalized local diversity index *vs.* the normalized global diversity index of HER2 expression in HER2-positive tumors. (**B**) Same as in (**A**), but for ER-positive tumors. Dashed lines, linear regression fits. ρ, Spearman correlation coefficient. (**C**) Mean normalized local *vs.* normalized global diversity of HER2 expression in HER2-positive cases. Horizontal black bars represent the mean of all cases in the corresponding group. The difference between the two groups is not statistically significant. (**D**) Same as in (**C**), but for ER. The *P* value was obtained with the Mann-Whitney test (two-tailed). (**E**) Mean normalized local diversity of HER2 expression in HER2-positive and Luminal B/HER2-positive molecular subtypes. Horizontal black bars represent the mean of all cases in the

corresponding group. The *P* value was obtained with the Mann-Whitney test (two-tailed). (**F**) Mean normalized local diversity of ER expression in Luminal A and B molecular subtypes. Horizontal black bars represent the mean of all cases in the corresponding group. The *P* value was obtained with the Mann-Whitney test (two-tailed).

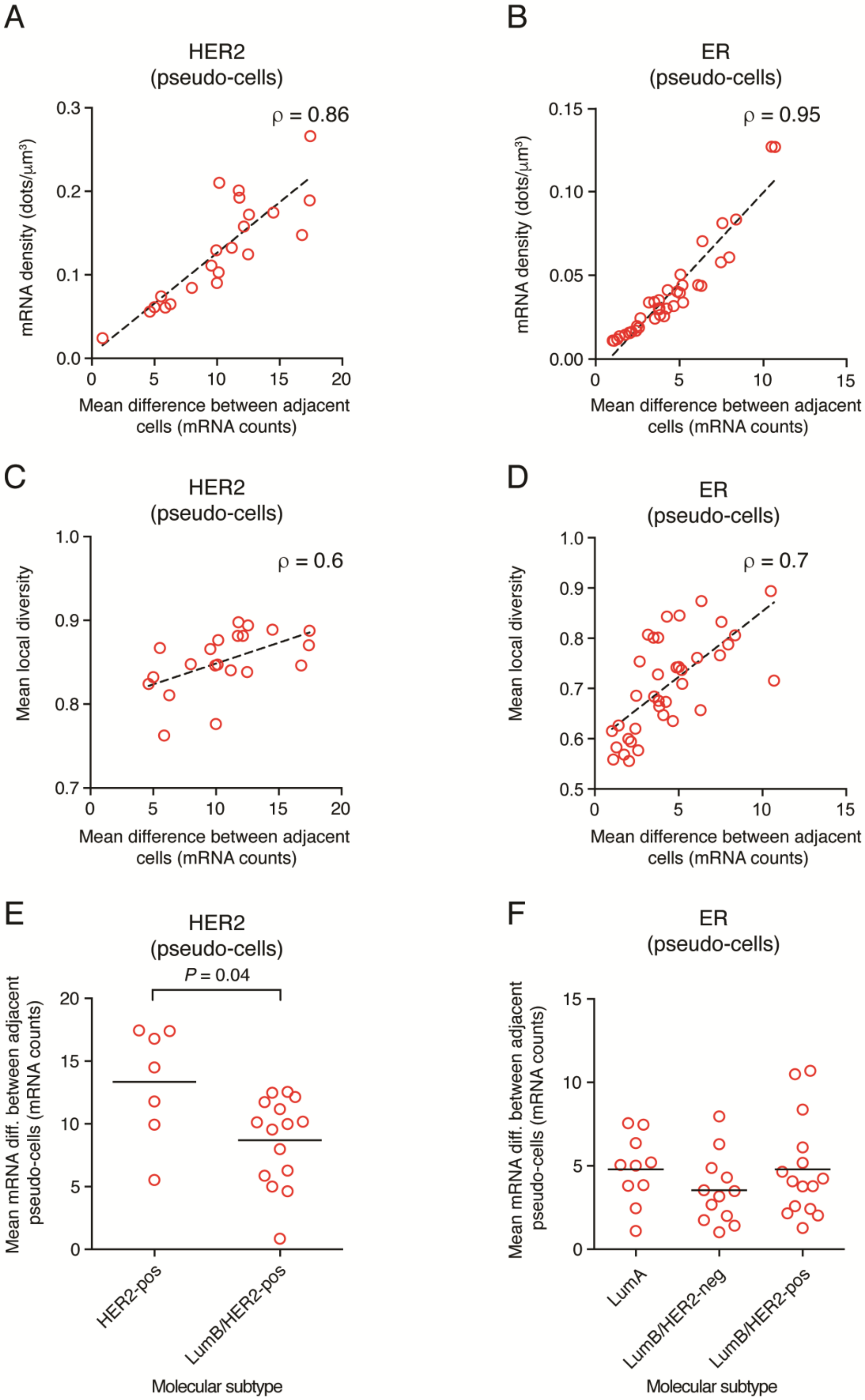
A
HER2
(pseudo-cells)
ρ = 0.86
mRNA density (dots/μm³)
Mean difference between adjacent cells (mRNA counts)
B
ER
(pseudo-cells)
ρ = 0.95
mRNA density (dots/μm³)
Mean difference between adjacent cells (mRNA counts)
C
HER2
(pseudo-cells)
ρ = 0.6
Mean local diversity
Mean difference between adjacent cells (mRNA counts)
D
ER
(pseudo-cells)
ρ = 0.7
Mean local diversity
Mean difference between adjacent cells (mRNA counts)
E
HER2
(pseudo-cells)
P = 0.04
Mean mRNA diff. between adjacent pseudo-cells (mRNA counts)
HER2-pos
LumB/HER2-pos
Molecular subtype
F
ER
(pseudo-cells)
Mean mRNA diff. between adjacent pseudo-cells (mRNA counts)
LumA
LumB/HER2-neg
LumB/HER2-pos
Molecular subtype

Supplementary Figure 7. (**A**) Spearman correlation between the mean difference in HER2 transcript counts among adjacent pseudo-cells and the mean HER2 transcript density per pseudo-cell in HER2-positive cases. Dashed line, linear regression fit. ρ, Spearman correlation coefficient. (**B**) Same as in (**A**), but for ER in ER-positive tumors. (**C**) Spearman correlation between the mean difference in HER2 transcript counts among adjacent pseudo-cells and the mean local diversity index of HER2 expression in HER2-positive tumors. Dashed line, linear regression fit. ρ, Spearman correlation coefficient. (**D**) Same as in (**C**), but for ER in ER-positive tumors. (**E**) Mean difference of HER2 transcript counts between adjacent pseudo-cells in HER2-positive versus Luminal B/HER2-positive molecular subtypes. (**F**) Same as in (**E**), but for ER in Luminal A and B subtypes.

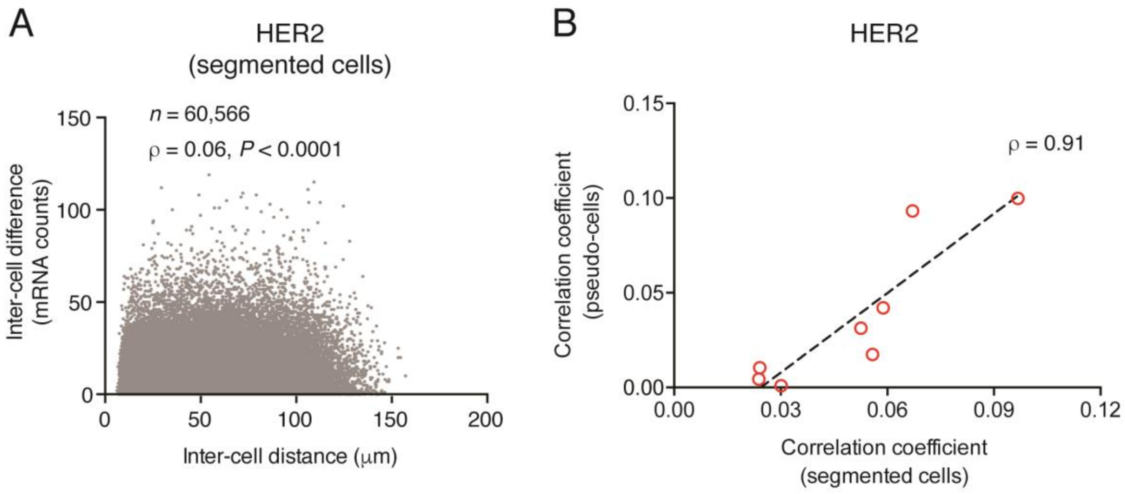

Supplementary Figure 8. (**A**) Pair-wise Spearman correlation between the physical distance separating all pairs of segmented cells in the same field of view and the absolute HER2 transcript count difference between the same pair of cells. *n*, number of cell pairs analyzed in HER2-positive cases. (**B**) Comparison of Spearman correlation between the physical distance among all cell pairs in the same field of view and the absolute HER2 transcript count difference, for segmented *vs.* pseudo-cells in 8 HER2-positive and Luminal B/HER2-positive tumors manually segmented. Dashed line, linear regression fit. ρ, Spearman correlation coefficient.

FFPE-smFISH step-by-step protocol

REAGENTS

- RBS™ 35 solution (Sigma, cat. no. 83461)
- Poly-L-lysine solution 0.1% (w/v) (Sigma #P8920)
- Xylene histological grade (Sigma cat.no. 534056-4L-D)
- Ethanol absolute (VWR cat. no. 20821.31)
- Methanol CHROMASOLV®, for HPLC, ≥99.9% (Sigma, cat. no. 34860-1L-R)
- Acid acetic (Sigma, cat. no. 695092-500ML-D)
- Nuclease-free water (Ambion, cat. no. AM9932)
- Nuclease-free Phosphate-Buffered Saline (10X) pH 7.4 (Thermo, cat. no. AM9625)
- RNaseZap® RNase Decontamination Solution (Ambion, cat. no. AM9784)
- Sodium citrate (SCBT, cat. no. sc-203383)
- Pepsin from porcine gastric mucosa (Sigma, cat. no. P6887-250MG)
- Hydrochloric acid, ACS reagent 37% (Sigma, cat.no. 258148-25ML-D)
- Sodium borohydride ($NaBH_4$) powder ≥98.0% (Sigma, cat. no. 452882-5G)
- Dextran sulphate (Sigma, cat. no. D8906)
- Formamide (Ambion, cat. no. AM9342)
- *E. coli* tRNA (Sigma, cat. no. R4251)
- Bovine Serum Albumin (Ambion, cat. no. AM2616)
- Ribonucleoside Vanadyl Complex (RVC) (NEB, cat. no. S1402S)
- 20X SSC buffer (Ambion, cat. no. AM9765)
- UltraPure™ 1 M Tris-HCI Buffer, pH 7.5 (Thermo, cat. no. 15567027)
- D-(+)-Glucose ≥99.5% (GC) (Sigma, cat. no. G8270)
- RNA*secure*™ RNase Inactivation Reagent (Ambion, cat. no. AM7006)
- 4′,6-Diamidino-2-phenylindole dihydrochloride (Sigma, cat. no. D8417-1MG)

CONSUMABLES

- VistaVision™ Cover Glasses No.1 (VWR, cat. no. 16004-098)
- Corning™ Costar™ Microcentrifuge Tubes (Fisher, cat. no. 07200210)
- Secure-Seal™ Hybridization Chambers (EMS, cat. no. 70333)
- Disposable scalpels, Minor (VWR, cat. no. SWAN2373)
- Fixogum (Leica Microsystems AB, cat. no. LK071A)

EQUIPMENT

- 20-Slide Unit Staining Dish Set (EMS, cat. no. 70312-20)
- EasyDip™ Slide Staining Jar (EMS, cat. no. 71385-W)
- EasyDip™ Slide Staining Rack (EMS, cat. no. 71386-DR)
- High Precisions and Ultra Fine Tweezers, Length: 4¼" (EMS, cat. no. 78320-5B)

COVER GLASS COATING

- Using a small piece of tape, attach the coverglasses to be washed each onto a regular microscope slide
- Place the slides into a staining dish pre-filled with 200 ml 2% RBS 35
- Sonicate 15 min in a sonication bath
- Thoroughly rinse the coverglasses under running ultrapure water
NOTE: do not pour water directly onto the coverglasses to avoid detaching them
- Fill the staining dish with 200 ml Ethanol 100%
- Sonicate 15 min
- Thoroughly rinse the coverglasses under running ultrapure water
NOTE: do not pour water directly onto the coverglasses to avoid detaching them
- Fill the staining dish with 200 ml Ethanol 100%
- Thoroughly rinse the coverglasses under running ultrapure water
NOTE: do not pour water directly onto the coverglasses to avoid detaching them
- Fill the staining dish with 200 ml Poly-L-lysine 0.01% (freshly prepared in nuclease-free water)
- Incubate 30 min at room temperature
- Dry the coverglasses by gently wiping a side of the holding slide using an absorbing paper
- Let the coverglasses dry overnight at room temperature, protected from dust
- Store the coverslips at room temperature up to 2 weeks inside a vacuum chamber

BUFFER PREPARATION

Hybridization buffer (HYB) (volumes for 10 ml)

- Weight 1 g of dextran sulfate in an RNAse-free manner in a 1.5 ml RNAse-free tube
- Add 4 ml of nuclease-free water and 1 ml of 20X SSC buffer into a 15 ml RNAse-free tube
- Gradually add the dextran powder (add a bit of powder at a time and vortex; continue until all the powder has been added)
- Rotate at rt until all the powder is dissolved. Meanwhile, thaw an RVC aliquot in a water bath pre-warmed at 70 °C until no sediment is visible
- Centrifuge briefly to remove the liquid from the lid
- Add 500 μl of RVC to the dissolved dextran. If a precipitate forms, place the tube at 70 °C for 2-3 min until dissolved
- Centrifuge at ~4000 g for 5 min and if there is a pellet of precipitated RVC, transfer the supernatant to a new tube (perform this centrifugation even if you didn't observe the precipitation in the previous step)
- Add 40 μl of BSA solution, 500 μl of tRNA solution, and 2.5 ml of formamide
-Rotate the solution for 5-10 min, and centrifuge briefly to remove liquid from the lid
- Aliquot and store at –20 °C

Wash buffer (WASH) (volumes for 50 ml)

- In a 50ml RNAse-free tube mix the following reagents:

1. 20X SSC buffer 5 ml
2. Deionized formamide 12.5 ml
3. Nuclease-free water 32.5 ml

- Mix and store protected from light at +4°C

NOTE: the solution can be stored up to 6 months. Equilibrate at room temperature before opening the tube

Equilibration buffer (EQ) (volumes for 10 ml)

- In a 15ml RNAse-free tube mix the following reagents:

1. 20X SSC buffer 1 ml
2. Tris buffer pH 7.5 100 μl
3. 40% glucose 100 μl
4. Nuclease-free water 8.8 ml

- Mix and store at +4 °C

NOTE: the solution can be used up to 1 week. Prepare the glucose solution freshly with nuclease-free water and treat it with RNA Secure according to the manufacturer's instructions.

Anti-bleach buffer (GLOX) (volumes for 100 μl)

-In a 1.5ml RNAse-free tube mix the following reagents:

1. TROLOX 5 μl
2. Glucose oxidase 1 μl
3. Catalase 1 μl
4. Nuclease-free water 93 μl

- Mix and use immediately

NOTE: prepare the solution freshly just before imaging

STEP-BY-STEP PROCEDURE

NOTE: up to 8 tissue sections can be processed in parallel using the following protocol

Day 1

Baking

- Attach one coverslip with a mounted FFPE section onto a microscope slide taping the edge close to the smeared slide area, where the sample name is written
- Bake the tissue for 16 h at 56 °C

Day 2

NOTE: in the following steps, “Jar” denotes a step performed in a separate EasyDip™ Slide Staining Jar previously decontaminated with RNaseZap®. Unless otherwise indicated, all steps are done at room temperature

Deparaffinization

- Jar #1: Xylene, 10 min
- Jar #2: Xylene, 10 min
- Jar #3: Ethanol 100%, 5 min
- Jar #4: Ethanol 100%, 5 min
- Jar #5: Methanol-Acetic Acid 3:1 (v/v), 5 min
- Jar #6: Ethanol 100%, 3 min
- Jar #7: Ethanol 85%, 3 min
- Jar #8: Ethanol 70%, 3 min
- Jar #9: nuclease-free water, 3 min

RNA retrieval

- Jar #10: Sodium citrate 0.01 M pH 6 + RVC 1:20 pre-warmed at 80 °C
-Incubate 45 min at 80 °C in a water bath
- Jar #11: nuclease-free water, 3 min
- Jar #12: Ethanol 70%, 3 min
- Jar #13: Ethanol 85%, 3 min
- Jar #14: Ethanol 100%, 3 min
-Air-dry tissue sections at room temperature
NOTE: at this point, tissue sections can be stored dry for several days at +4 °C
-Cover each tissue section with a Secure-Seal™ Hybridization Chamber
NOTE: the chamber that we use (EMS, cat. no. 70333) can fit approximately 100 μl. All steps below are done by injecting and aspirating 105 μl in/out of the chamber, at room temperature unless otherwise stated

Pepsin digestion

- Ethanol 100%, 5 min
- Ethanol 85%, 3 min
- Ethanol 70%, 3 min
- Nuclease-free water, 3 min
- Pepsin 0.025% in Hydrochloric acid 0.01 M, 15 min
- Nuclease-free water, 3 min

Autofluorescence quenching

- Sodium borohydride, 15 min
NOTE: prepare 10 ml solution in 1X PBS on ice freshly each time. Exert extreme caution while weighing the powder, as accidentally dropping liquid in the stock bottle will result in an explosive reaction! Because gas bubbles form continuously in the chamber, aspirate the liquid and replenish the chamber with fresh solution every 1-2 min during the incubation
- After the incubation, repeatedly wash the chamber 4-5 times with nuclease-free water

Pre-hybridization

- 2X SSC buffer, 5 min
- WASH buffer, 5 min

Hybridization

- In a 1.5ml RNAse-free tube mix the following reagents:
  1. HYB buffer 100 µl
  2. smFISH probe 60-300 ng

NOTE: the HYB buffer must be equilibrated at room temperature prior to each use. The optimal amount of probe varies and must be determined empirically for each probe
- Vortex at maximum force for 5 sec and then spin down
- Add the hybridization mix to the chamber
- Seal the chamber with Fixogum
-Incubate 16-18 h in an incubator at 30 °C

Day 3

Washing

- Aspirate the hybridization mix from the chamber and rinse the chamber 2-3 times with WASH buffer pre-warmed at room temperature
- Incubate in WASH buffer for 90 min at 30 °C
- Exchange to WASH buffer + DAPI 70 ng/ml
- Incubate for 30 min at 30 °C
- 2X SSC, 5 min
- EQ buffer, 2 min

Imaging

OPTION 1 (for tissue sections directly attached onto microscope slides)
- Carefully remove the chamber from the slide using a scalpel and gently dry the leaked-out liquid with an absorbent paper, being careful not to touch the tissue
- Wet the tissue with approx. 100 µl of freshly prepared GLOX buffer
- Carefully place a coverslip on top of the tissue and remove the excess liquid with absorbent paper
- Seal the coverslip with Fixogum and image

OPTION 2 (for tissue sections attached onto coverglasses)
- Fill the Secure-Seal™ chamber with GLOX buffer
- Seal the chamber with Fixogum and mount a microscope slide onto the Fixogum (now the chamber lies in between the slide and the coverglass)
- Wipe the coverglass with ethanol 100% and image
